# A Biologically Plausible Computational Theory for Value Integration and Action Selection in Decisions with Competing Alternatives

**DOI:** 10.1371/journal.pcbi.1004104

**Published:** 2015-03-24

**Authors:** Vassilios Christopoulos, James Bonaiuto, Richard A. Andersen

**Affiliations:** 1 Division of Biology and Biological Engineering, California Institute of Technology, Pasadena, California, United States of America; 2 Sobell Department of Motor Neuroscience and Movement Disorders, University College London, London, United Kingdom; Northwestern University, United States of America

## Abstract

Decision making is a vital component of human and animal behavior that involves selecting between alternative options and generating actions to implement the choices. Although decisions can be as simple as choosing a goal and then pursuing it, humans and animals usually have to make decisions in dynamic environments where the value and the availability of an option change unpredictably with time and previous actions. A predator chasing multiple prey exemplifies how goals can dynamically change and compete during ongoing actions. Classical psychological theories posit that decision making takes place within frontal areas and is a separate process from perception and action. However, recent findings argue for additional mechanisms and suggest the decisions between actions often emerge through a continuous competition within the same brain regions that plan and guide action execution. According to these findings, the sensorimotor system generates concurrent action-plans for competing goals and uses online information to bias the competition until a single goal is pursued. This information is diverse, relating to both the dynamic value of the goal and the cost of acting, creating a challenging problem in integrating information across these diverse variables in real time. We introduce a computational framework for dynamically integrating value information from disparate sources in decision tasks with competing actions. We evaluated the framework in a series of oculomotor and reaching decision tasks and found that it captures many features of choice/motor behavior, as well as its neural underpinnings that previously have eluded a common explanation.

## Author Summary

In high-pressure situations, such as driving on a highway or flying a plane, people have limited time to select between competing options while acting. Each option is usually accompanied with reward benefits (e.g., avoid traffic) and action costs (e.g., fuel consumption) that characterize the value of the option. The value and the availability of an option can change dynamically even during ongoing actions which compounds the decision-making challenge. How the brain dynamically integrates value information from disparate sources and selects between competing options is still poorly understood. In the current study, we present a neurodynamical framework to show how a distributed brain network can solve the problem of value integration and action selection in decisions with competing alternatives. It combines dynamic neural field theory with stochastic optimal control theory, and includes circuitry for perception, expected reward, effort cost and decision-making. It provides a principled way to explain both the neural and the behavioral findings from a series of visuomotor decision tasks in human and animal studies. For instance, the model shows how the competitive interactions between populations of neurons within and between sensorimotor regions can result in “spatial-averaging” movements, and how decision-variables influence neural activity and choice behavior.

## Introduction

From very simple decisions, such as selecting what to wear or choosing a place for dinner, to more complex decisions, such as trading in the stock market, we usually have to select among competing options. Selecting between alternatives requires assigning and integrating values along a multitude of dimensions with different currencies, like the energetic cost of movement and monetary reward. Solving this problem requires integrating disparate value dimensions into a single variable that characterizes the “attractiveness” of each option. In dynamic decisions, in which the environment changes over time, this multi-dimensional integration must be updated across time. Despite the significant progress that has been made in understanding the mechanisms underlying dynamic decisions, little is known on how the brain integrates information online and while acting to select the best option at any moment.

A classic psychological theory, known as “goods-based decision making”, posits that decision making is a distinct cognitive function from perception and action and that it entails assigning values to the available goods [1–5]. According to this theory, multiple decision determinants are integrated into a subjective economic value at the time of choice. The subjective values are independently computed for each alternative option and compared within the space of goods, independent of the sensorimotor contingencies of choice. Once a decision is made, the action planning begins. This view is in accordance with evidence suggesting the convergence of subjective value in the orbitofrontal cortex (OFC) and ventromedial prefrontal cortex (vmPFC), where the best alternative is selected (for a review in “goods-based” theory see [4]). Despite the intuitive appeal of this theory, it is limited by the serial order assumption. Although many economic decisions can be as simple as choosing a goal and pursuing it, like choosing between renting or buying a house, humans evolved to survive in hostile and dynamic environments, where goal availability and value can change with time and previous actions, entangling goal decisions with action selection. Consider a hypothetical hunting scenario, in which a predator is faced with multiple alternative valuable goods (i.e., prey). Once the chase begins, both the relative value of the goods and the cost of the actions to pursue these goods will change continuously (e.g., a new prey may appear or a current prey may escape from the field), and what is currently the best option may not be the best or even available in the near future. In such situations, the goals dynamically compete during movement, and it is not possible to clearly separate goal decision-making from action selection.

Recent findings argue against a purely “goods-based” theory for decisions between actions, suggesting that decisions are made through a continuous competition between partially prepared action-plans. According to this “action-based” theory, when the brain is faced with multiple goals, it initiates concurrent and partially prepared action-plans that compete for selection and uses value information accumulated during ongoing actions to bias this competition, until a single goal is pursued [6–11]. This theory received support from neurophysiological [8, 12–15] and behavioral [9, 10, 16–21] studies, and it is in accord with the continuous flow model of perception, which suggests that response preparation can begin even before the goal is fully identified and a decision is made [22–24]. However, it is vague as to how action costs are dynamically integrated with good values and other types of information.

In the current study, we propose a distributed neurodynamical framework that models the neural basis of decision-making between actions. It allows dynamic integration of value information from disparate sources, and provides a framework that is rich enough to explain a broad range of phenomena that have previously eluded common explanation. It builds on successful models in dynamic neural field theory [25] and stochastic optimal control theory [26] and includes circuitry for perception, expected reward, selection bias, decision-making and effort cost. We show that the complex problem of action selection in the presence of multiple competing goals can be decomposed into a weighted mixture of individual control policies, each of which produces optimal action-plans (i.e., sequences of actions) to pursue particular goals. The key novelty is a relative desirability computation that dynamically integrates value information to a single variable, which reflects how “desirable” it is to follow a policy (i.e., move in a particular direction), and acts as a weighting factor on each individual policy. Because desirability is state- and time- dependent, the weighted mixture of policies automatically produces a range of behavior, from winner-take-all to weighted averaging. Another important characteristic of this framework is that it is able to learn sensorimotor associations and adapt the choice behavior to changes in decision values of the goals. Unlike classic neurodynamical models that used hard-wired associations between sensory inputs and motor outputs, we allow for plasticity in the connections between specific dynamic neural fields (DNFs) and, using reinforcement learning mechanisms, we show how action-selection is influenced by trained sensorimotor associations or changing reward contingencies.

By integrating dynamic neural field theory with control theory, we developed a biologically plausible framework that explains many aspects of human and animal behavior and its neural underpinnings in decisions between actions. It provides insights to a variety of findings in neurophysiological and behavioral studies, such as the competitive interactions between populations of neurons within [27, 28] and between [29] brain regions that result frequently in spatial averaging movements [10, 18, 30] and the effects of decisions variables on neuronal activity [31, 32]. We also make novel predictions concerning how changes in reward contingencies or introduction of new rules (i.e., assigning behavioral relevance to arbitrary stimuli) influence the network plasticity and the choice behavior.

## Results

### Model architecture

**Fig 1:**
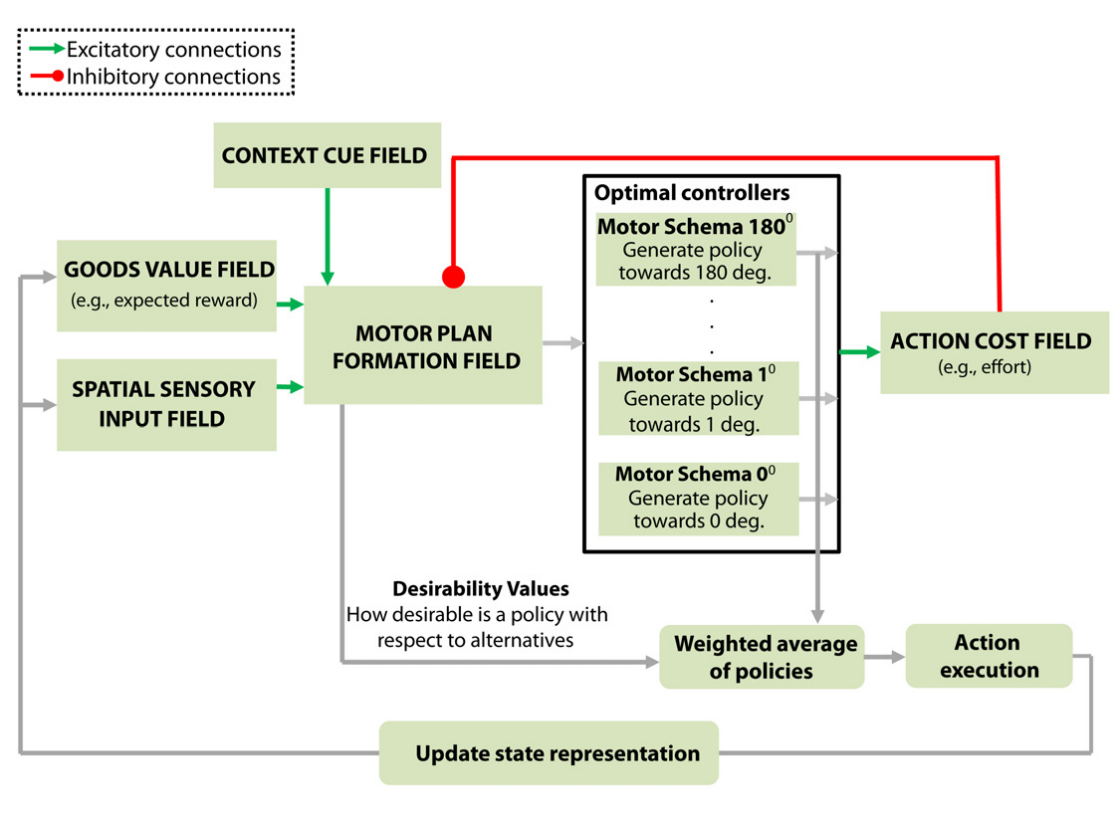
Model architecture. The core component of the model is the motor plan formation field that dynamically integrates information from disparate sources. It receives excitatory inputs (green lines) from: i) the spatial sensory input field that encodes the angular representation of the alternative goals, ii) the goods-value field that encodes the expected benefits for moving towards a particular direction and iii) the context cue field that represents information related to the contextual requirements of the task. The motor plan formation field also receives inhibitory inputs (red line) from the action cost field that encodes the action cost (e.g., effort) required to move in a particular direction. All this information is integrated by the motor plan formation field into an evolving assessment of the “desirability” of the alternative options. Each neuron in the motor plan formation field is linked with a motor control schema that generates a direction-specific policy *π*_*j*_ to move in the preferred direction of that neuron. The output activity of the motor plan formation field weights the influence of each individual policy on the final action-plan (see “Model architecture” in the results section for more details).

The basic architecture of the framework is a set of dynamic neural fields (DNFs) that capture the neural processes underlying cue perception, motor plan formation, valuation of goods (e.g., expected reward/punishment, social reward, selection bias, cognitive bias) and valuation of actions (e.g., effort cost, precision required), Fig. 1. Each DNF simulates the dynamic evolution of firing rate activity within a neural population. It is based on the concept of population coding, in which each neuron has a response tuning curve over some set of inputs, such as the location of a good or the end-point of a planned movement, and the responses of a neuronal ensemble represent the values of the inputs. The functional properties of each DNF are determined by the lateral interactions within the field and the connections with the other fields in the architecture. Some of these connections are static and predefined, whereas others are dynamic and change during the task. The projections between the fields are topologically organized, that is, each neuron in one field drives activation of the corresponding neuron (coding for the same direction) in the fields to which it projects.

Let’s consider the hunting scenario, in which a predator is faced with multiple goods (i.e., prey) located at different distances and directions from the current state **x**_*t*_. The architectural organization of the present framework to model this type of problem is shown in Fig. 1. The “spatial sensory input field” encodes the angular spatial representation of the alternative goods in an egocentric reference frame. The “goods value field” encodes the goods values of pursuing each prey animal irrespective of sensorimotor contingencies (e.g., effort). The “context cue field” represents information related to the contextual requirements of the task. The outputs of these three fields send excitatory projections to the “motor plan formation field” in a topological manner. Each neuron in the motor plan formation field is linked with a motor control schema that generates both a direction-specific optimal policy *π*_*i*_, which is a mapping between states and best-actions, and an action cost function *V*_*π*_*i*__ that computes the expected control cost to move in the direction *ϕ*_*i*_ from any state (see Methods section and S1 text for more details). It is important to note that a policy is not a particular sequence of actions—it is rather a function that calculates the best action-plan **u**_*i*_ (i.e., sequences of actions/motor commands) to take from the current state **x**_*t*_ to move in the direction *ϕ*_*i*_ for *t*_*end*_ time-steps (i.e., *π*_*i*_(**x**_*t*_) = **u**_*i*_ = [*u*_*t*_, *u*_*t*+1_, …, *u*_*t*_*end*__]).

The neurons in the motor plan formation field with activation levels above a threshold, *γ*, trigger the associated motor schemas. Once a motor schema is active, it generates a policy *π*(**x**_*t*_) towards the preferred direction of the corresponding neuron. The associated action cost is encoded by the action cost field. The “action cost field” in turn inhibits the motor plan formation field via topological projections. Therefore, the role of the motor plan formation field is two fold: i) trigger the motor schemes to generate policies and ii) integrate information associated with actions, goals and contextual requirements into a single value that characterizes the “attractiveness” of each of the available policies. The output of the motor plan formation field encodes what we call the *relative desirability* of each policy and the activity of the field is used to weigh the influence of each policy in the final action. As soon as the chase begins, the action costs and the estimates of the values related to the goals will change continuously—e.g., a new prey may appear at the field modulating the relative reward of the alternatives. The advantage of our theory is that it integrates value information from disparate sources dynamically while the action unfolds. Hence, the relative desirability of each policy is state- and time- dependent and the weighted mixture of policies produces a range of behavior from winner-take-all selection of a policy to averaging of several policies.

### Visuomotor decisions with competing alternatives

The general framework described above can be translated into more concrete and testable scenarios, such as visuomotor decision tasks with competing goals and/or effectors for comparison with experimental data. We show how the proposed computational model can be extended to involve motor plan formation DNFs for various effectors which interact competitively to implement effector as well as spatial decision-making.

#### Action selection with multiple goals

**Fig 2:**
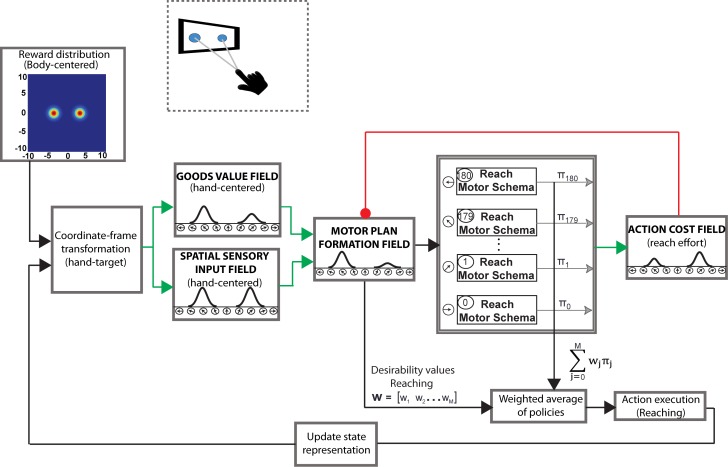
A simplified version of the model architecture for reaching decision tasks in the presence of two competing targets. The motor plan formation field encodes the direction of the intended arm movement in a special reference frame centered on the hand. The goods-based decision values and the spatial sensory inputs are therefore transformed from allocentric to egocentric representations centered on the hand before being input to the motor plan formation field. The motor plan formation field integrates information about the spatial location of the targets, the expected reward attached to each target and the action cost required to pursue the targets into a single variable named “relative desirability”. The relative desirability encodes the “attractiveness” of the individual *M* reach policies at a given time and state and is used to weigh the influence of these policies on the final policy. Note that *M* is the number of neurons with activation level above a threshold *γ*. Once the final policy is determined, the framework implements that policy at the given time and state resulting in an action-plan (i.e., sequences of actions) that drives the hand closer to the target (see Results and Methods sections for more details).

In the current section, we present a simplified version of the framework that involves only reaching, Fig. 2. The reach motor plan formation DNF encodes the direction of intended arm movements in a spatial reference frame centered on the hand. Various decision values are therefore transformed from allocentric to egocentric representations centered on the hand before being input to the reach motor plan formation DNF. The DNF receives input encoding the location of the stimulus, the expected reward associated with moving in each direction and the action cost. The location of each stimulus is encoded in the spatial sensory input field as a Gaussian population code centered on the direction of the stimulus with respect to the hand. The expected reward for moving to given locations is effector-independent and encoded as a multivariate Gaussian population in allocentric coordinates. This representation is transformed into a one dimensional vector in the goods value field representing the expected reward for moving in each direction, centered on the hand position. These values, as well as the action cost field inputs, are summed and applied to the reach motor plan formation DNF. The DNF is run until it reaches a peak activity level above *γ*, after which its values are used to weigh the policy of each motor schema, resulting in a reach movement.

**Fig 3:**
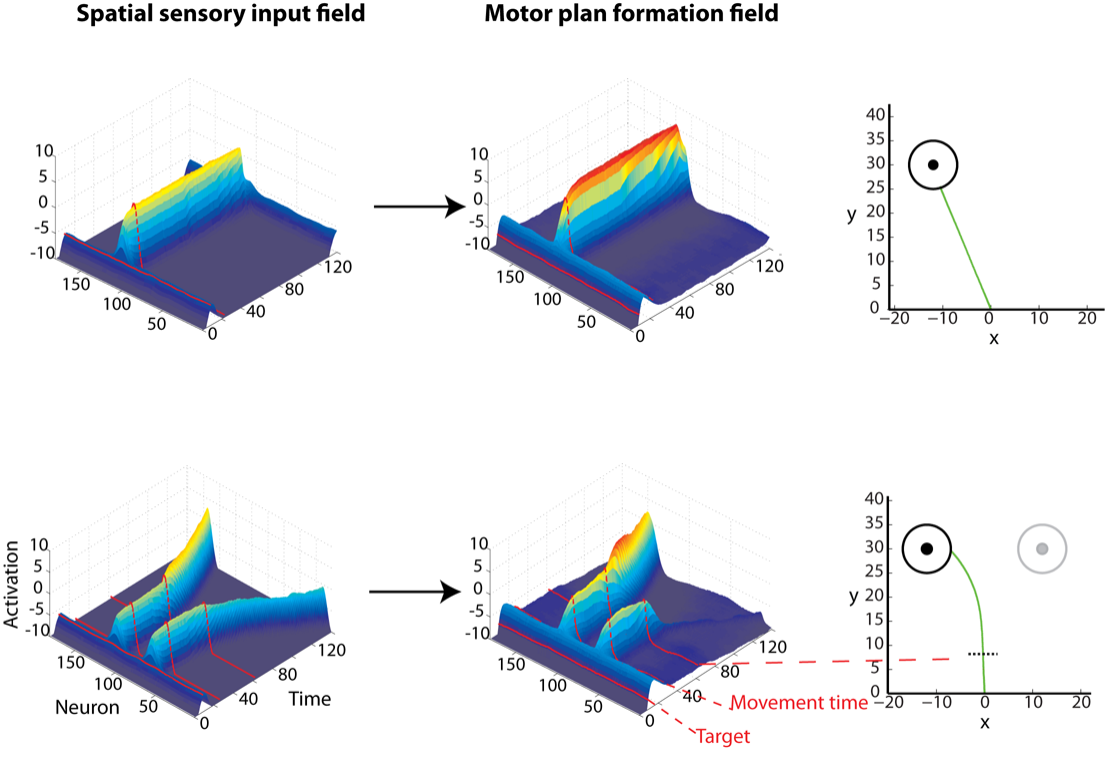
Characteristic examples of the simulated model activity in single- and two- target trials with the corresponding reaching movements, for a “rapid reaching choice” experiment. Each trial started with either a single black target or two gray potential targets presented simultaneously in both visual fields. In the single-target trials, the actual target location was known prior to reach onset (upper row). In the two-target trials, reaching movements were initiated without knowing the actual location of the target (bottom row). Each of the two potential targets had an equal probability to turn black (i.e., cued target). When the hand crossed a pre-defined threshold (black discontinuous line in the bottom right panel), the actual target was cued for action. The left and the middle columns depict the activity in the spatial sensory input field and the motor plan formation field for the single- (top row) and two- (bottom row) target trials. Notice that when both potential targets were presented, the activity in the motor plan formation field was weaker, compared to activity generated in a single-target trials where the actual location of the target was known in advance. The populations of neurons related to the two potential targets remained active and competed against each other until the actual target was cued, and the activity of the non-cued target vanished. On trials when only a single target is presented, the reaches were made directly to the target. However, when two potential targets are presented in the field, the model generates spatial averaging reaching movements—i.e., initial reaching movement towards an intermediate position between the two targets followed by a corrective movement to the cued target. “Movement time” (or response time) indicates the time that the framework initiated the reaching movement.

The operation of the framework can be easily understood in the context of particular reaching choice tasks that involve action selection in the presence of competing targets. We designed and simulated an experiment that involves rapid reaching movements towards multiple potential targets, with only one being cued after movement onset [9, 10, 18–21]. We implemented the rapid responses required in this task by reducing the activation threshold *γ*, so that the controllers are triggered shortly after the targets are displayed. In some of these trials, a single target was presented—the model knew in advance the actual location of the target. The results showed that the present framework can reproduce many characteristics of neuronal activity and behavior reported in the experimental literature. Particularly, the presence of two potential targets caused an increase of the activity of two ensembles of neurons, each of them tuned to one of the targets, Fig. 3. Importantly, the motor plan formation DNF activity was weaker compared to activity generated in single-target trials (i.e., no uncertainty about the actual location of the target), due to competitive field interactions, Fig. 3. When one of the targets was cued for action, the activity of the corresponding neuronal ensemble increased, while the activity of the non-cued target was reduced. These findings are consistent with neurophysiological studies on hand reaching tasks, showing the existence of simultaneous discrete directional signals associated with the targets in sensorimotor regions, before making a decision between them [7, 8, 13, 27, 33]. The neuronal activity was weaker while both potential targets were presented prior to the reach onset, compared to the single-target trials [27, 33].

The present framework also makes novel predictions on how the neuronal response patterns evolve over time after the reach onset. In particular, in the two-target trials, the neuronal ensembles in the motor plan formation field remained active and competed against each other for a period of time, until the actual target was cued, and the activity of the non-cued target vanished, Fig. 3. Finally, the competition between the two populations of neurons caused spatial averaging movements, as opposed to straight reaches in the single-target trials, Fig. 3. This finding is in agreement with experimental studies, which showed that when multiple potential targets are presented simultaneously, and participants are forced to rapidly act upon them without knowing which one will be the actual target, they start reaching towards an intermediate location, before correcting the trajectories “in-flight” to the cued target location [9, 10, 16, 18–21]. In a similar manner, the present framework can be easily extended to model other rapid reaching choice experiments, such as eye and hand movements made to targets in the presence of non-target visual distractors [16, 34, 35].

#### Action selection with competing effectors

So far, we have focused on visuomotor decision tasks with competing targets and shown that decisions emerge through mutual inhibition of populations of neurons that encode the direction of movements to the targets. However, the motor system is inherently redundant and frequently the brain has to solve another competition problem—which effector to use to pursue an action. Neurophysiological studies in non-human primates have shown that when the animals are presented with a target, which can be pursued either by reaching or saccades, both the parietal reach region (PRR) and lateral intraparietal area (LIP), which are specialized for planning reaching and saccadic movements, respectively, are simultaneously active before an effector is selected [29, 36]. Once an effector is chosen, the neuronal activity in the corresponding cortical area increases, while the activity in the area selective for the non-selected effector decreases. Similar findings have also been reported in human studies for arm selection in unimanual reaching tasks [37]. These results suggest that a similar mechanism for action selection with competing goals also accounts for selecting between competing effectors.

We modeled this type of visuomotor decision task, in which a target can be acquired either with a reach or eye movement, by duplicating the architecture of the framework described in the previous section and designating one network for saccades and one for reaches. Input to the saccade network was encoded in eye-centered coordinates, while input to the reach network was encoded in hand-centered coordinates. Each motor plan formation DNF also received inhibitory projections from every neuron in the other motor plan formation DNF, implementing effector selection in addition to the spatial selection performed by each DNF in isolation. The context cue field encoded the task context with half of its neurons responding to a saccade cue and half responding to a reach cue. This layer was fully connected with each motor plan formation DNF, with weights initially randomized with low random values and trainable through reinforcement learning (S1 Fig. in supporting information shows analytically the architecture of the framework for effector choice tasks).

**Fig 4:**
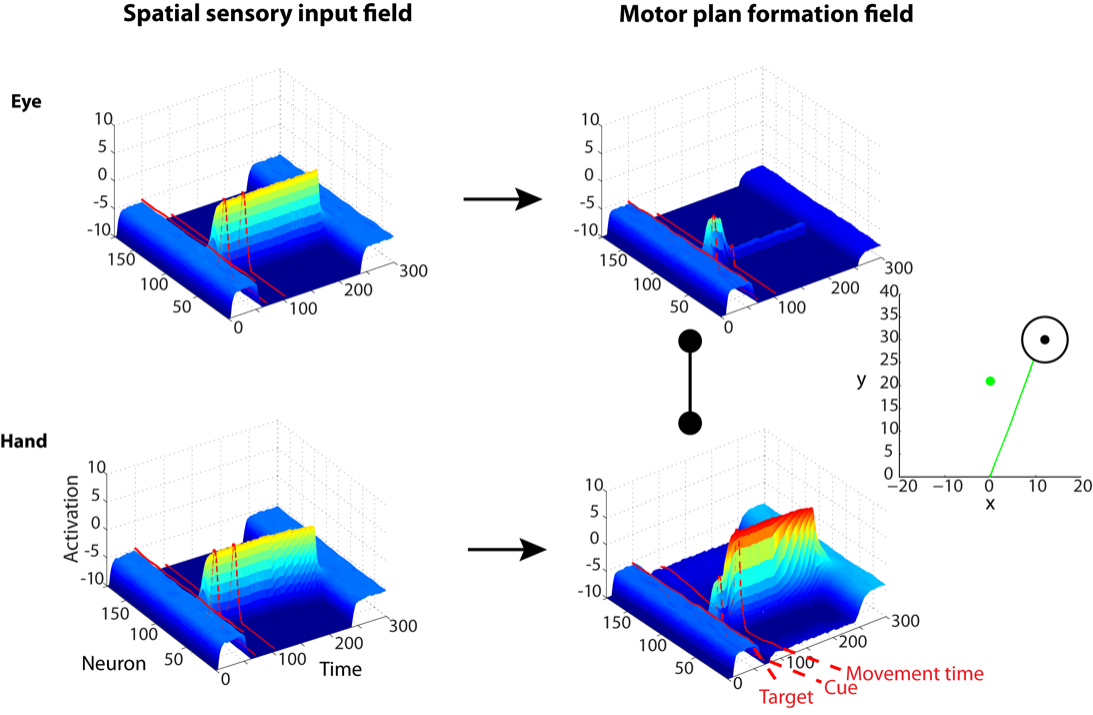
Characteristic example of the simulated model activity during an effector choice task. A single target, which can be acquired with either a hand or an eye movement, is presented at about 50 time-steps after the trial onset. The activity of the neurons tuned to this target increases in both DNFs that plan hand and eye movements, since the framework does not know whether it is a “reach” or a “saccade” trial. Once the “green” cue is presented about 20 time-steps after the target onset (the hypothetical (x,y) location of the green cue is represented by the green dot in the Cartesian plot to the right panel), the neuronal activity in the reaching DNF (bottom right field) becomes sufficiently strong, due to the excitatory inputs from the context cue neurons, to inhibit the saccade DNF (upper right field). The competition is resolved shortly and the framework generates a direct reaching movement (green trace) to the target.

Let’s assume for now that the weights of the context cue neurons are already learned, such that the framework knows that “red” and “green” cues indicate saccades and reaches, respectively. Fig. 4 depicts a trial from an “effector-decision” task with a single target similar to the neurophysiological study described above [29]. A target is presented 50 time-steps after the trial onset, followed by a “green” cue signal 20 time-steps later. Consistent with the experimental findings, once the target is presented, the activity of the populations of neurons tuned to the direction of the target increases in the motor plan formation DNFs for both reaching and saccadic movements, since the model does not yet know whether it is a “reach” or “saccade” trial. Notice that during that time, the neuronal activity in the reach DNF is lower than the activity in this field for the single target trial with no effector competition (see Fig. 3 upper panel), due to the inhibitory interactions with the motor plan formation DNF for saccadic movements. Once the green cue is presented, the neuronal activity in the reach DNF becomes strong enough to inhibit the saccade DNF and conclusively wins the competition, and the model generates a direct reaching movement to the target.

**Fig 5:**
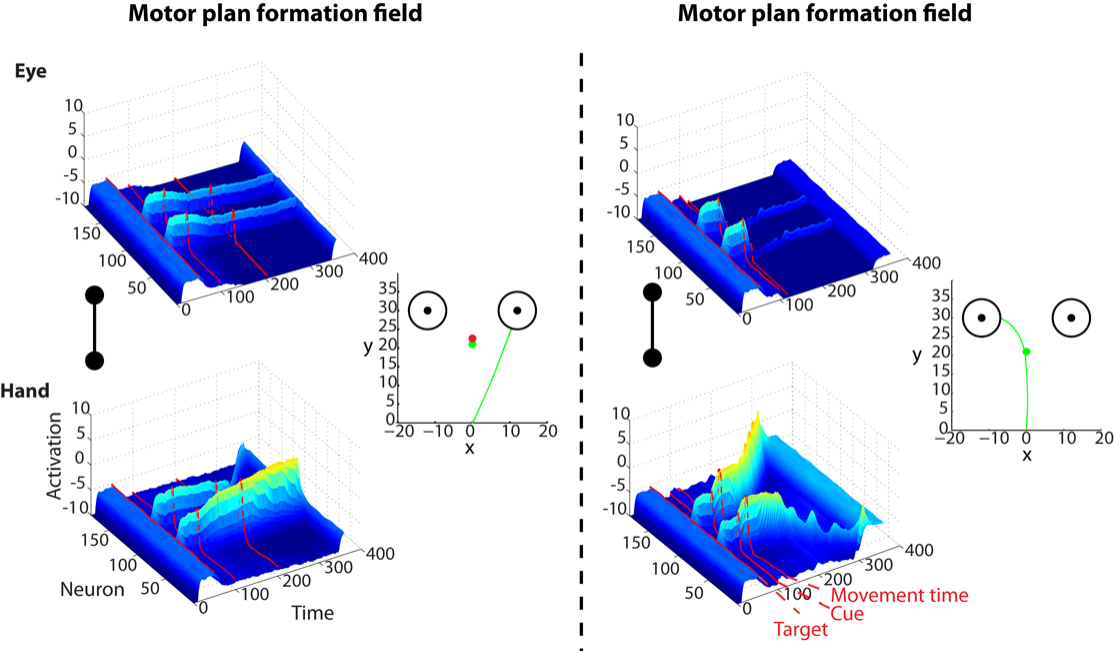
Characteristic example of the simulated model activity during an effector choice task with two targets. *Left*: Neuronal activity of the DNFs that plan saccade (upper row) and reaching (bottom row) movements in a free-choice task with two competing targets. Two targets located in the left and the right hemifield at equal distance from the “hand” and “eye” origin are presented at 50 time-steps followed by a “free-choice” cue signal (red and green cues are presented simultaneously) 50 time-steps later, which indicates that the framework is free to choose any effector to acquire any of the two targets. Since there is no effector preference to bias the effector competition, it takes longer for the model to decide whether to use the “hand’ or the “eye” to acquire the target. As a result, the competition between the targets is usually resolved before the movement onset, resulting frequently in direct movements to the selected target (green trace is a characteristic example of reaching movement in a free-choice trial). *Right*: Similar to the left panel but for a “cued-reaching” trial (green cue). The effector competition is resolved shortly after the cue is presented and the movement starts sooner than the free-choice trial due to the excitatory inputs from the context cue neurons. Thus, the competition between the targets is usually not resolved before the movement onset resulting in curved trajectories (green trace is a characteristic example of reaching movement in a “cued-reaching” trial).

**Fig 6:**
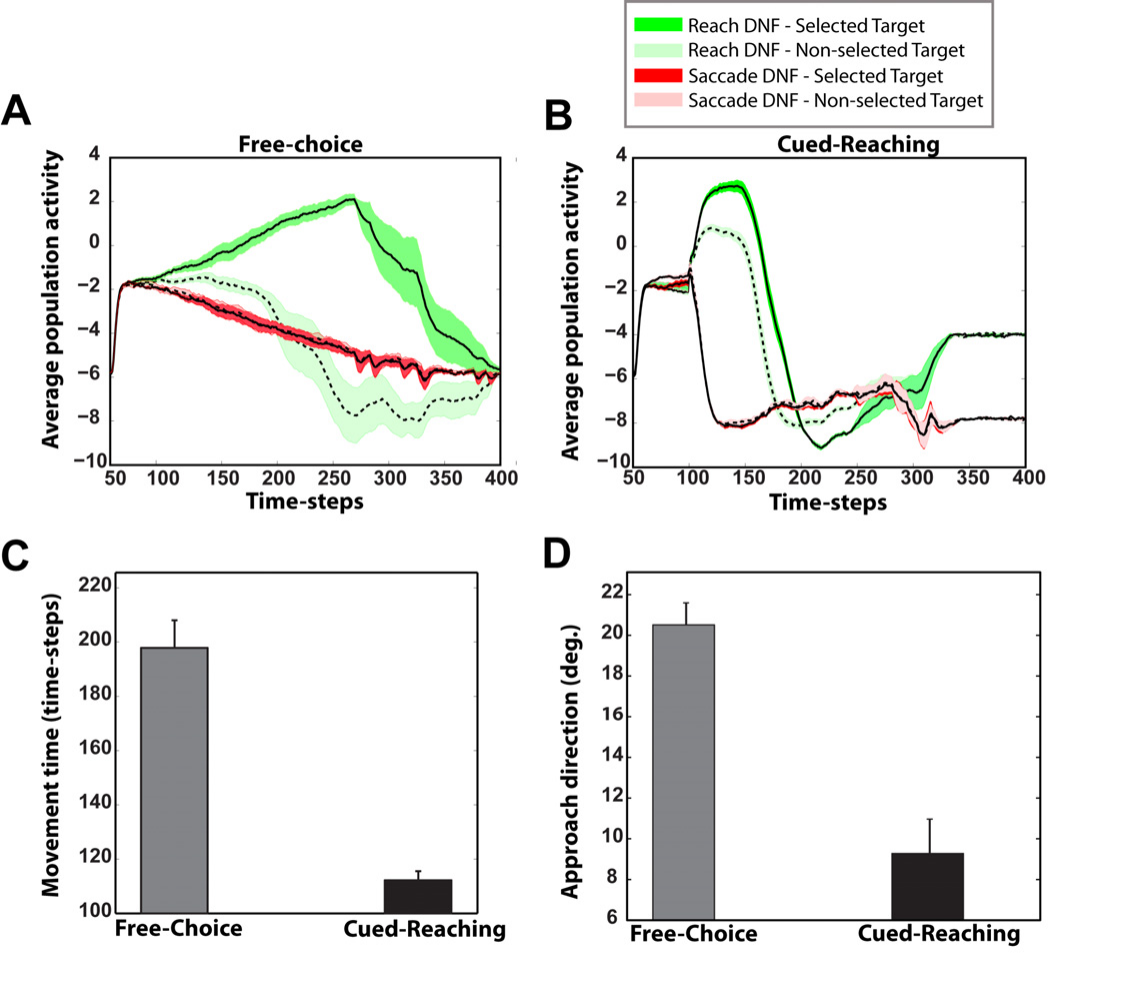
Simulated neural activity, movement time and approach direction of reaching trajectories, in free-choice and cued-reaching trials in an effector choice task. **A**: Time course of the average activity (20 trials) of the two populations of neurons tuned to the selected (solid black line) and the non-selected (discontinuous black line) targets prior to movement onset, from the DNFs that plan reaching (green color) and saccade (red color) movements in the “free-choice” sessions. Data shown only when reaches were selected. Notice that the framework selects first which effector to use to perform the task and then it chooses the target. The average activity from the saccade DNF for the selected and non-selected targets overlaps. **B**: Similar to panel A, but for the “cued-reaching” sessions. The competition between the effectors is resolved almost immediately after the cue onset. **C**: Mean movement (i.e., response) time from 20 reaching trajectories in a free-choice task (i.e., model is free to choose to perform hand or eye movement to acquire each of the targets) and a cued task, in which the model was instructed to perform reaches. The error bars are ± standard error. The movement time in the free-choice trials was significantly lower than the movement time in the cued-reaching trials (two-sample *t*-test, *p* < 10^−7^). **D**: Mean approach direction of 20 reaching movements for the first 50 time-steps in a free-choice task and a cued-reaching task. The error bars are ± standard error. Approach direction at 0 deg. indicates that initial reaching movements were made towards the intermediate location between the two targets. Notice that free-choice trials are characterized with straight reaching movements to the selected target, whereas the cued-reaching trials are dominated mostly by curved reaching movements to the selected target (two-sample *t*-test, *p* < 10^−4^).

These results suggest that effector selection utilizes a similar mechanism to action selection with multiple target goals. This raises the question of how the brain may solve a competition that involves both multiple goals and effectors. To address this question, we designed and simulated a novel visuomotor decision task that involves multiple competing targets which can be acquired by either saccadic or reaching movements. To the best of our knowledge, this task has not been studied yet with experiments in humans or animals. The left and right panels in Fig. 5 illustrate the simulated neural activity in motor plan formation DNFs for eye and hand movements for a characteristic trial in “free-choice” and ‘cued-reaching” sessions, respectively. In the free-choice condition, two equally rewarded targets are presented in both hemifields 50 time-steps after the beginning of the trial followed by a free-choice cue (i.e., red and green cues simultaneously presented on the center of the screen) 50 time steps later. In this condition, the framework is free to choose either of the two effectors to acquire any of the targets. In the cued condition, a green or a red cue is presented 50 time steps after the onset of the targets, indicating which effector should be used to acquire either of the targets. There are several interesting features in the simulated neuronal activities and behavior:

- The competition between the effectors is solved earlier than the competition between the targets for both cases. This finding is shown better in Fig. 6A and B that depict the average activity of the two populations of neurons tuned to the selected (solid black lines) and the non-selected (discontinuous black lines) targets from both DNFs that plan reaching (green) and eye (red) movements in the free-choice and cued-reaching sessions, respectively. Notice that in both sessions, the framework chooses first which effector to use (i.e., hand or eye) and then it selects which target to acquire. This is explained by the massive inhibitory interconnections between the DNFs that plan reaches and saccades.
- The effector competition takes more time to be resolved in free-choice trials than in cued trials.
- Because it takes longer to decide which effector to use in free-choice trials, the competition between the two targets is usually resolved before the movement onset, frequently resulting in direct reaching or saccadic movements to the selected target (the green trace in the left panel of Fig. 5 is a characteristic example of a reaching trajectory from free-choice trials).
- In the cued trials, the competition between the effectors is resolved shortly after the cue onset. Once the cue appears, the activity of the neurons which are tuned to both targets in the motor plan formation DNF of the cued effector increases, inhibiting the neurons in the motor plan formation DNF of the non-cued effector. However, the excitation of the populations of neurons in the DNF of the cued effector amplifies the spatial competition which selects a particular target (see also Fig. 6B). Frequently this competition is not resolved before the movement onset, resulting in curved trajectories (i.e., spatial averaging movements) (see the green trace in Fig. 5 right panel).

We further quantified the effects of multiple effectors and multiple targets in action selection by computing the movement onset (i.e., response time) and the direction of 20 trajectories for the first 50 time-steps after the movement onset in both “free-choice” and “cued” trials (50 time-steps was selected somewhat arbitrarily, although it corresponds to about 1/3 of the time to contact the target). The results illustrated in Fig. 6C and D show that free-choice trials are characterized by slower response times and straighter trajectories, whereas cued trials are characterized by faster response times and more highly curved trajectories to the selected targets.

**Fig 7:**
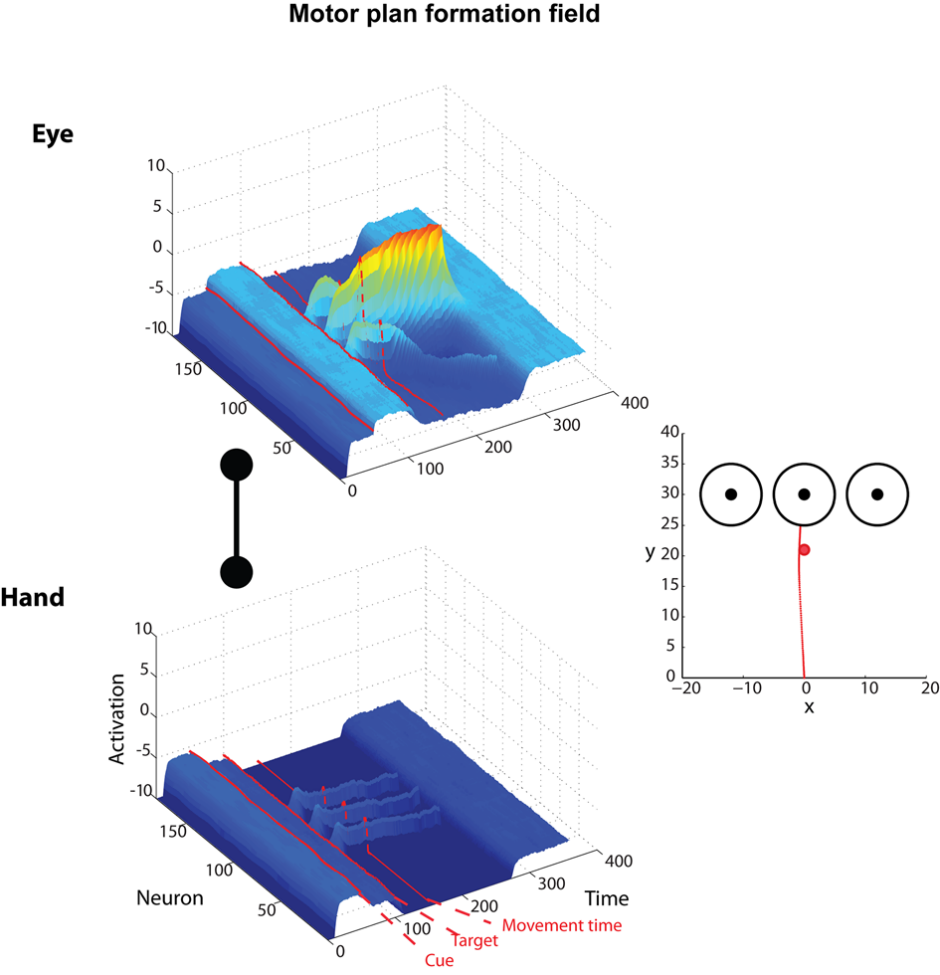
Characteristic example of the simulated model activity during an effector choice task with three targets. Neuronal activity of the DNFs that plan saccade (upper row) and reaching (bottom row) movements during a “cued-saccade” trial (note the red cue), in which the context cue is presented prior to target onset. The competition between the effectors is resolved shortly after the context cue is presented. Once the locations of the targets are shown, the framework has already selected the effector (i.e., eye in this trial) and the competition between the targets is resolved quite fast resulting in direct saccadic movements to the selected target (right panel).

Finally, we designed and simulated another novel experiment with competing goals and effectors, but this time the effector cue was presented before the targets. In such a scenario, the motor plan formation DNF for the effector associated with the given cue received enough input to almost completely inhibit the other DNF. Once the targets appeared, the decision activity proceeded exclusively in the DNF for the cued effector and the only thing that remained to be resolved was the competition between the targets. Fig. 7 depicts such a scenario with 3 targets, in which the cue is presented 50 time steps after the trial starts, followed by the target onset 50 time steps later. Notice that the effector competition is resolved almost immediately after cue onset. All of these findings are novel and have not been validated in human or animal experimental studies, suggesting new avenues to understand the neural and behavioral mechanisms of action-selection in decisions with competing alternatives.

#### Sensorimotor association learning in effector choice tasks

Although the present framework makes qualitative predictions of many features of neuronal activity and behavior in an effector choice task, it assumes that the brain knows *a priori*, the association between color cues and actions. How the brain learns sensorimotor associations remains poorly understood. We studied the neural mechanisms underlying sensorimotor association learning by training the model to distinguish between two cues which signal whether it is required to perform a reach (“green” cue) or saccade (“red” cue) to a target. During each trial, a cue was presented, followed by a target randomly presented in one of three positions. The context cue field with half of its neurons selective for the reach cue and half for the saccade cue was fully connected with each motor plan formation DNF. These weights were updated using reinforcement learning following every trial, *T*, with a reward given for moving to a target with the cued effector:

$$\begin{array}{c} W_{cue}\left(T+1\right)=W_{cue}\left(T\right)+\alpha_{cue}r\left(T\right)u_{cue}\left(T\right)E_{effector}\left(T\right) \end{array}$$ (1)

Where **W**_*cue*_ is the weight matrix for connections between the cue-selective population and the motor plan formation DNF for the effector, *α*_*cue*_ is the learning rate, *r* is the reward signal (1 if the task was successfully performed, 0 otherwise), **u**_*cue*_ is the vector of cue-selective neuron activity, and **E**_*effector*_ is the eligibility trace for the effector (a decaying copy of the associated motor plan formation DNF activity) [38].

**Fig 8:**
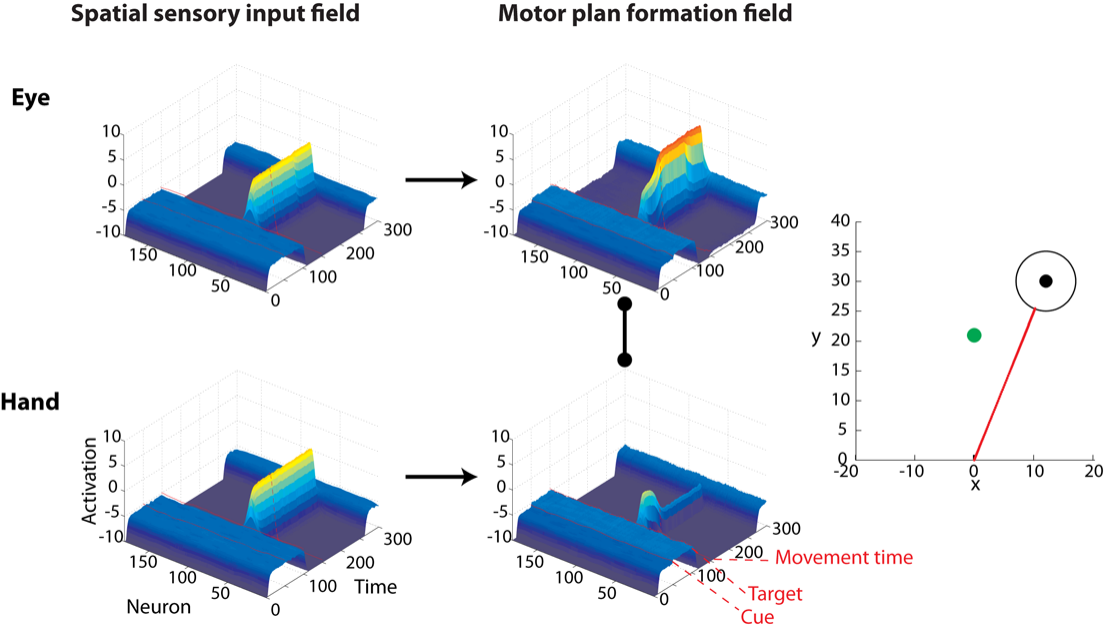
Characteristic example of the simulated model activity during training with a reach cue presented first, followed by a single target. Stimulus input activity (left column) and motor plan formation DNF activity (middle column) for the eye (top row) and hand (bottom row) networks. The model incorrectly performed a saccade in response to the reach cue (right column).

**Fig 9:**
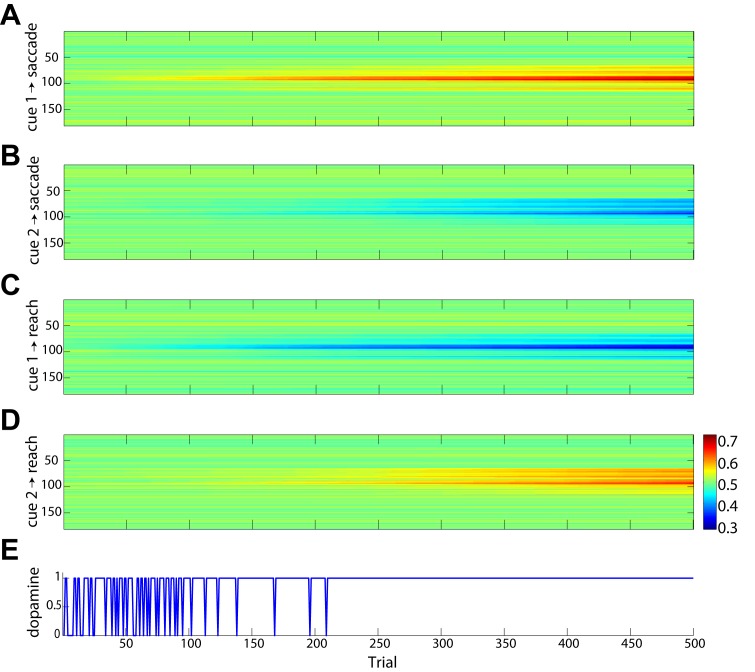
History of training on effector cues. Plots A-D show the connection weights from neurons representing each cue (i.e., red and green) to the saccade (A-B) and reach (C-D) motor plan formation DNFs. There are 50 neurons selective for each cue and each motor plan formation field has 181 neurons, yielding four 50×181 connection weight matrices. Each matrix has been averaged over the cue selective neurons at each trial to show the mean connection weight to each motor plan formation field as training progresses. **A**: Mean connection weights from neurons representing the red cue (cue 1) to neurons in the saccade motor formation DNF from trials 1 to 500. **B**: Mean connection weights from green cue (cue 2) neurons to the saccade DNF. **C**: Mean connection weights from red cue neurons to the reach motor formation DNF. **D**: Mean connection weights from green cue neurons to the reach motor formation DNF. **E**: Success of each trial during training (0 = unsuccessful, 1 = successful).

During the learning period, when the context cue connections were not fully trained, the model would frequently perform actions with the wrong effector. A characteristic example of incorrect trials during the learning period is shown in Fig. 8. The “green” cue is presented at about 50 time-steps after the trial starts, increasing the activity in both DNFs that plan saccadic and reach movements, because the framework is still learning the sensorimotor associations. Once the target appears, the DNF that forms the eye movements wins the competition and the model performs a saccade, although a “green” cue is presented. The evolution of the context cue connection weights during training is shown in Fig. 9A-D. Fig. 9A shows the average connection weights between the red cue (cue 1) population and saccade motor plan formation DNF as training progressed for 500 trials. Similarly, Fig. 9B shows the mean connection weights from the green cue (cue 2) neurons to the saccade motor plan formation, and Fig. 9C and 9D show the mean connection weights from the red cue and the green cue populations to the reach motor plan formation DNF. After just over 200 training trials, the model learned the sensorimotor associations and its performance reached 100% (Fig. 9E).

#### The effects of decision variables on action selection

Thus far, we have considered only cases in which the competing targets are equally rewarded for correct choices. However, people frequently make decisions between alternative options with different values. In the current section, we show how decision variables, such as the expected reward attached to a target, influence choice/motor behavior and its neural underpinnings in decisions with multiple competing alternatives. We designed and simulated a reaching experiment that included choices between two targets which appeared simultaneously in the left and right hemifields at equal distance from the starting hand position. Both targets appeared 50 time-steps after the trial onset and had either the same expected reward (“equal-reward” trials) or one of them had higher expected reward than the other (“unequal-reward” trials).

**Fig 10:**
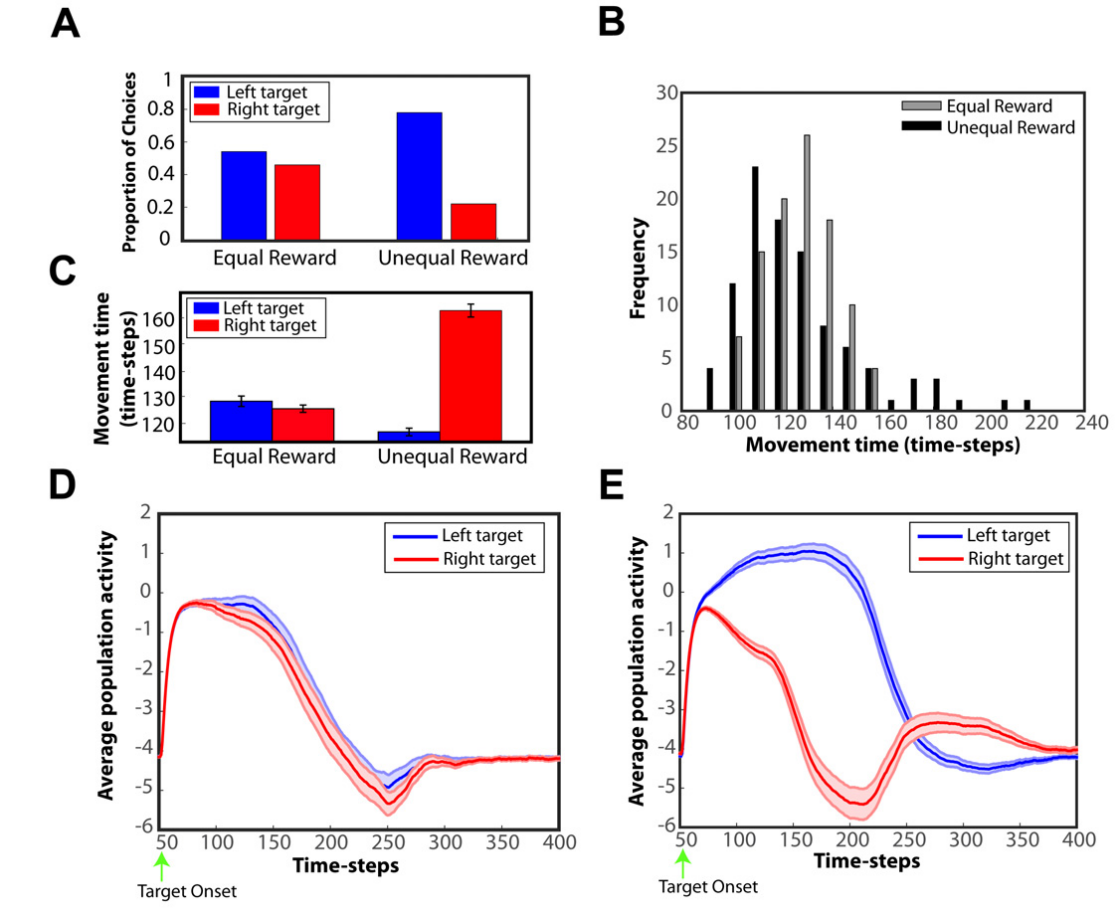
Expected reward biases the competition between alternative actions. **A**: Proportion of left and right choices in the “equal-reward” and “unequal-reward” conditions. Expected reward influences choice preferences by shifting the choice bias towards the higher valued target. **B**: Movement time distribution for “equal-reward” (gray bars) and “unequal-reward” (black bars) choices. The movement time is approximately normally distributed for equal-reward choices, and it becomes increasingly skewed to the right for unequal-reward choices. **C**: Average movement time for reaches to the left and right target in the equal-reward and unequal-reward conditions. The error bars are ± standard error. Notice that reach responses become faster when choosing the most preferred option than when selecting the less preferred option (two-sample *t*-test, *p* < 10^−6^). **D**: Time course of the average activity of the two populations of neurons tuned to the targets from the DNF that plans the reaches in the “equal-reward” condition. The target onset is indicated by a green arrow. The temporal evolution and the strength of the neural activity are about the same for both populations, since the expected reward for both choices is the same. **E**: Similar to panel D, but for the “unequal-reward” condition. In this case, the modulation of the expected reward influences the neural activity in the reach DNF—the activity of neurons tuned to the higher valued target increases significantly compared to the neuronal activity associated with the lower valued target.

The results showed that reward expectancies have a profound effect both on choice behavior and motor behavior. Particularly, we found that choices were biased towards the higher valued target, and the movement time was significantly lower when choosing the most preferred option over the other in the two-target trials with unequal expected reward. To illustrate this, consider a scenario where two targets are presented simultaneously in both visual fields, and the left target has 3 times higher expected reward than the right one in the unequal-reward trials. Fig. 10A depicts the proportion of choices to the left and the right target, in both equal-reward and unequal-reward conditions. Notice the significant choice bias to the higher valued target in the unequal-reward choices. Fig. 10B illustrates the distribution of the movement time after 100 trials for equal-reward (gray bars) and unequal reward (black bars) choices. The movement time distribution is approximately Gaussian when choices are made between equally rewarded options. However, it becomes increasingly skewed to the right in unequal reward choices (two-sample Kolmogorov-Smirnov test, *p* = 0.0131). We also computed the average movement time for selecting the left and the right target both in equal-reward and unequal-reward trials. The results presented in Fig. 10C show that the movement time was about the same when selecting either target in the equal-reward choices (two-sample *t*-test *p* = 0.3075). However, the movement time significantly decreased when choosing the most favored option over then less favored option in the unequal-reward trials (two-sample *t*-test *p* < 10^−6^). Similar results were found for saccade choices (results are not shown here for the sake of brevity). These predictions have been extensively documented in a variety of visuomotor tasks, which showed that reward expectancy modulates both the choice and the motor behavior. Particularly, when subjects had to decide among options with different expected reward values, the choices were more likely to be allocated to the most rewarded option [32, 39]. Moreover, psychophysical experiments in humans and animals showed that the response time (i.e., movement time) is negatively correlated with the expected value of the targets [39–41].

The reward expectancies also modulated the neural activity of the populations of neurons tuned to these targets in the reaching motor plan formation DNF. Fig. 10D and E illustrate the time course of the average activity of the two populations of neurons from 100 trials in the “equal-reward” and “unequal-reward” conditions, respectively. When both targets are associated with the same expected reward, both populations have about the same average activity across time resulting in a strong competition for selecting between the two targets and hence no significant choice bias and slower movement times. On the other hand, when one of the targets provides more reward than the other, the average activity of the neuronal ensemble selective for that target is significantly higher than the activity of the neurons tuned to the alternative target. Thus, the competition is usually resolved faster, resulting frequently in a selection bias towards to the most valuable target and faster movement time. Occasionally, the neuronal population in the reach DNF, which is selective to the target with the lower expected reward, wins the competition, and the lower valued target is selected. However, it takes more time on average to win the competition (i.e., longer movement time), because it receives weaker excitatory inputs from the goods-value field compared to the neuronal ensemble, which is selective for the higher valued target (see S2 Fig. in supporting information for more details.) These findings are consistent with a series of neurophysiological studies in non-human primates, which showed that decision variables, such as the expected reward, reward received over a recent period of time (local income), hazard rate and others modulate neurons in parietal cortex and premotor cortex both in reaching and saccade tasks [12, 32, 42–44]. The proposed computational framework suggests that this effect occurs due to the excitatory inputs from the goods value field that encodes the goods-related decision variables. When one of the alternative options is associated with higher expected reward, the “goods value” neurons that are tuned to this target have higher activity than the neurons related to less valuable options. Hence, the competition is biased towards to the alternative associated with greater reward. Note that similar findings can be predicted by the present framework for effort-based decisions, when one of the alternatives requires more effort to be acquired than the rest of the options. Particularly, the framework predicts that when two equally rewarded targets are simultaneously presented in the field, the choices are biased towards the “less-expensive” (i.e., less effort) target, and reaching movements become faster when they are made to that target (results are not shown here for the sake of brevity).

#### Reward contingency learning

In the previous analysis, we assumed that the framework has already learned the values of the alternative options before taking any action. However, in many conditions, people and animals have to explore the environment to learn the values of the options. In the current section, we present the mechanism that the framework uses to learn reward contingencies. Each motor plan formation DNF received input signalling the expected reward for moving to a particular location. The expected reward was represented in a two-dimensional neural field, **U**_*reward*_, in allocentric coordinates and converted to an egocentric representation in the goods value field centered on the appropriate effector before being input to each motor plan formation DNF (see Methods section). The activity in the field **U**_*reward*_ was computed by multiplying a multivariate Gaussian encoding stimulus position with a set of weights, **W**_*reward*_, which were also updated using reinforcement learning following every trial *T*:

$$\begin{array}{c} W_{reward}\left(T+1\right)=W_{reward}\left(T\right)+\alpha_{reward}r\left(T\right)E_{spatial}\left(T\right) \end{array}$$ (2)

where *α*_*reward*_ is the learning rate, *r* is the reward signal (1 or 0), and **E**_*spatial*_ is the eligibility trace. In this case the reward is effector-independent, so the eligibility trace simply encodes the location of the last movement (whether reach or saccade) as a multivariate Guassian.

**Fig 11:**
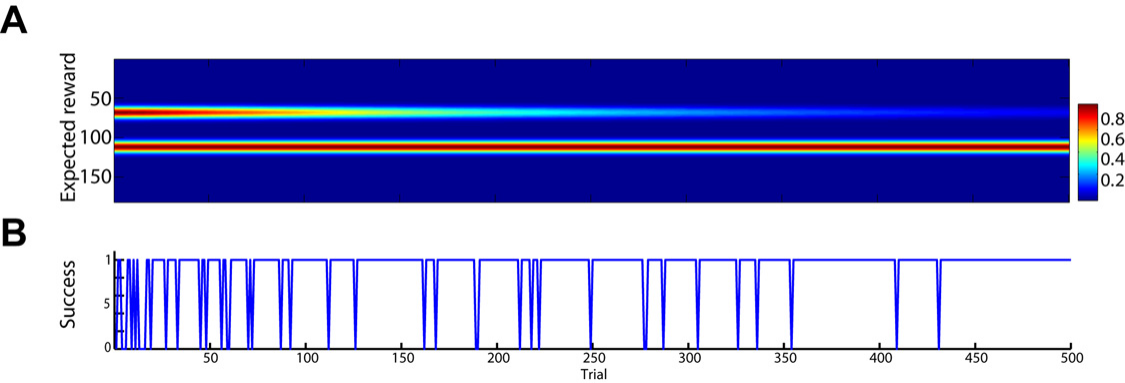
History of training on reward contingency. **A**: Expected reward for target directions in an egocentric reference frame from trials 1 to 500. The model was presented with two targets on each trial, initialized with equal expected reward. Reward was received for reaching or making a saccade to the left target. **B**: Success of each trial during training (0 = unsuccessful, 1 = successful).

We tested the model by presenting two targets and no context cue, but rewarding it whenever it made a reach or saccade to the left target. The evolution of the weights, **W**_*reward*_, over 500 training trials is shown in Fig. 11A converted to a two-dimensional egocentric frame. The weights projecting to neurons representing each target were initialized with equal levels of expected reward. After approximately 300 training trials, the weights to neurons encoding the right target decreased enough to reach almost 100 percent accuracy (Fig. 11B). Because the expected reward signal was broadcast to both motor plan formation DNFs, the model made both reaching and saccade movements at equal frequency.

## Discussion

One of the most fundamental questions in decision neuroscience is how the brain selects between alternative options. Classic serial theories, such as the “goods-based” model, suggest that decision-making is an independent cognitive process from the sensorimotor processes that implement the resulting choice [1]. According to this view, decisions are made in higher cognitive centers by integrating all the decision values of an option into a subjective value and selecting the best alternative option. Once a decision is made, the sensorimotor system implements the selected choice [2–4]. Clinical and neurophysiological studies have shown that a neural representation of subjective values exists in certain brain areas, most notably in the orbitofrontal cortex (OFC) and ventromedial prefrontal cortex (vmPFC) [3, 45].

Although the “goods-based” theory can explain a variety of decisions, such as choosing a place to have dinner tonight, it is limited by the serial-order assumption. This raises the question of how we decide between competing actions in dynamic environments, where the value and the availability of an option can change unpredictably. One possibility that has been suggested by a number of researchers is that decisions between actions are made through a continuous competition between concurrent action-plans related to the alternative goals [8, 11, 13, 46]. According to this “action-based” theory, the competition is biased by decision variables that may come from higher cognitive regions, such as the frontal cortex, but the sensorimotor system is the one that decides which action to select [8, 15]. This view has received apparent support from series of neurophysiological studies, which found neural correlates of decision variables within sensorimotor brain regions (for review, see [7, 13, 15]). Additionally, recent studies in non-human primates have shown that reversible pharmacological inactivation of cortical and sub-cortical regions, which have been associated with action planning or multisensory integration, such as lateral intraparietal area (LIP) [47, 48], superior colliculus (SC) [49], and dorsal pulvinar [50], cause decision biases towards targets in the intact visual field. These findings suggest that sensorimotor and midbrain regions may be causally involved in the process of decision-making and action-selection.

While these experimental studies have contributed significantly to understanding the mechanisms underpinning decisions between multiple actions, they are framed using theories that are insufficient to describe, much less predict, existing findings. Several computational frameworks exist that explain how information from disparate sources, such as goods values, action costs, prior knowledge, and perceptual information, are integrated dynamically to evaluate and compare the available options [51–53]. However none of these generate continuous actions which change the desirability and availability of options as they unfold. Instead, they generate discrete choices and not population codes that can be used to guide action in continuous parameter spaces, and they do not capture the interactions between networks of brain regions in any realistic way (see below). In the current study, we propose a neurodynamical framework that models the neural mechanisms of value integration and action-selection in decisions with competing options. It is comprised of a series of dynamic neural fields (DNFs) that simulate the neural processes underlying motor plan formation, expected reward and effort cost. Each neuron in the motor plan formation DNF is linked with a stochastic optimal control schema that generates policies towards the preferred direction of the neuron. A policy is a function that maps the current state into optimal sequences of actions. The key novelty of the framework is that information related to goals, actions and contextual requirements is dynamically integrated by the motor plan formation DNF neurons and that this information changes and is re-evaluated as the action is performed. The current activity of each of these DNF neurons encodes what we call “relative desirability” of the alternative policies, because it reflects how “desirable” it is to follow a particular policy (i.e., move in a particular direction) with respect to the alternative options at a current state, and weights the influence of each individual policy in the final action-plan.

The present framework is related to classic “sequential sampling models”, such as the drift diffusion model (DDM) [54] and the leaky competing accumulator (LCA) [55], which suggest that selection between competing options involves the gradual accumulation of sensory evidence until a threshold is reached. Although the framework also employs an “accumulator” mechanism to dynamically integrate value information and decide between options, and an action threshold to determine when to trigger motor schemas, it is quite different from these classic models. The classic models assume that populations of neurons in particular brain areas accumulate the sensory evidence and other brain areas compare the accumulated evidence to make decisions. For instance, studies in oculomotor decision-making suggest that LIP neurons accumulate the evidence associated with the alternative options and the accumulated activity of these neurons is compared by “decision brain areas” to select an option [13, 56]. Unlike the classic models, in the present framework the decisions are made through a dynamic transition from “weighted averaging” of individual policies to “winner-take-all”—i.e., a set of policies associated with an available goal dominates the rest of the alternatives—within the same population of neurons in the motor plan formation field. It does not assign populations of neurons to individual options, rather the alternative options emerge within a distributed population of neurons based on the current sensory input and prior knowledge. Once any neuron reaches the action threshold its associated motor schema is activated, but the decision process continues and other motor schemas can become active during the performance of an action. Because of these characteristics, the present framework can handle not only binary choices, but also decisions with multiple competing alternatives, does not require a “decision” threshold (although it does use a pre-defined “movement initiation” threshold), and can model decisions in which subjects cannot wait to accumulate evidence before selecting an action; rather they have to make a decision while acting.

Additionally, the present framework shares many features with other systems-level computational frameworks that have been previously proposed to model decisions between multiple actions [57, 58]. However, these approaches do not incorporate the idea of dynamically integrating value information from disparate sources (with the exception of Cisek’s (2006) model [58], which demonstrated how other regions, including prefrontal cortex, influence the competition), they do not model action selection tasks with competing effectors, and they do not model the eye/hand movement trajectories generated to acquire the choices. By combining dynamic neural fields with stochastic optimal control systems the present framework explains a broad range of findings from experimental studies in both humans and animals, such as the influence of decision variables on the neuronal activity in parietal and premotor cortex areas, the effect of action competition on both motor and decision behavior, and the influence of effector competition on the neuronal activity in cortical areas that plan eye and hand movements.

The model presented here bears some similarity to decision-making models in the hierarchical reinforcement learning (HRL) framework [59]. HRL suggests that decision-making takes place at various levels of abstraction, with higher levels determining overall goals and lower levels determining sub-sequences of actions to achieve them. HRL does capture the dynamic aspect of decision-making shown by our model in that it re-evaluates all goals and selects the best current goal at each time point, but there are three major differences. The first is that HRL chooses a single policy and typically pursues it until all actions in a sequence are performed, whereas our model uses a weighted mixture of policies and has no notion of a fixed sequence of actions. The second is that HRL uses a softmax rule to choose a goal, while our model uses both the “goods-” and “action-based” signals. Third, while an HRL framework could be used in place of our model’s motor plan formation DNFs to select an effector and target, our model goes beyond that by interfacing this system with optimal motor control models to control the arm and eyes in real time.

Besides the predictions that have been already validated by experimental studies, the present framework makes novel predictions that have not yet been confirmed by any studies to the best of our knowledge. For instance, we predict that when the brain is faced with multiple equally rewarded goals and also has to select between competing effectors to implement the choice, it first resolves the effector competition (i.e., decides which effector to use) before selecting which goal to pursue. This effect is related to the inhibitory interactions within and between the fields that plan the movements. The motor plan formation fields are characterized by local excitatory and global one-to-many inhibitory connections. However, the interactions between the motor plan formation fields of the competitive effectors are characterized by global inhibitory interactions in which each neuron in one motor plan formation field inhibits all neurons in the other motor plan formation field, resulting in greater net inhibition between rather than within fields. It is because of this architecture that the effector competition is resolved prior to target competition. Although this prediction has not been tested in experimental studies, a recent fMRI study in humans showed that when people have to select whether to use the left or the right hand for reaching to a single target, the effector selection precedes the planning of the reaching movement in the dorsal parietofrontal cortex [37]. We should point out that this prediction is made when both targets have the same expected value and requires the same effort. By varying the value of the targets or the action cost, it is possible that the framework will first select the most “desirable” target, and then it will choose the best effector to implement the choice.

### Learning sensorimotor associations and reward contingencies

The present framework is able to learn appropriate behavior for tasks in which actions result in different levels of expected reward based on contextual cues or goal-based reward contingencies. This is due to reinforcement learning on connection weights between the goods value field and context cue field and the motor plan formation DNF, and simulates the process by which cortico-basal ganglia networks map contextual cues and reward expectancy onto actions [60]. An unexpected result of our simulations was that although the model was trained on the cued effector task with targets in multiple locations, it could only learn the task when it was presented with a single target in one of the possible locations on each trial, rather than multiple targets at once. This was due to interference between the sensorimotor association and reward contingency learning processes. As the model was rewarded for reaching to a particular target with the correct effector, it began to associate that target with reward in addition to the effector. While various parameter settings which increased the noise levels in the network would promote exploration and thus spatial generalization of the effector cue, the learning proceeded optimally when a single target was presented in one of many possible locations on each trial. This effect has been extensively documented in many studies and is known as “dual-task interference” [61]—when conflicting and interfering stream of information must be processed simultaneously in dual tasks (e.g., talking on the phone while driving), the performance of the tasks is deteriorated substantially.

### Mapping to neurophysiology

The computational framework presented in the current study is a systems-level framework aimed to qualitatively model and predict response patterns of neuronal activities in ensembles of neurons, as well as decision and motor behavior in action selection tasks with competing alternatives. It is not intended to serve as a rigorous anatomical model and because of this we avoid making any strict association between the components of the framework (i.e., individual DNFs and control schemes) and particular cortical and subcortical regions. However, it captures many features of neuronal activity recorded from different cortical areas such as the parietal reach region (PRR), area 5, lateral intraparietal area (LIP), premotor cortex, prefrontal cortex (PFC) and orbitofrontal cortex (OFC) in non-human primates that perform reaching and saccadic decision tasks with competing options.

This model can be conceived as a coarse-grained sketch of the fronto-parietal cortical network involved in decisions between competing actions. The “spatial sensory input field” encodes the spatial location of the targets in an egocentric reference frame and mimics the organization of the posterior parietal cortex (PPC). The “context cue field” represents information related to the task context (i.e., which effector to use to acquire the targets). Several neurophysiological studies have reported context-dependent neurons in the lateral PFC (LPFC) in non-human primates [62–64]. These neurons respond differently to the same stimulus when it requires different responses depending on the task context, whereas they are not sensitive to the color or pattern of the cue. The “goods value field” integrates the good values of the alternative options and represents how “desirable” it is to select a policy towards a particular direction without taking into account the sensorimotor contingencies of the choices (i.e., the action cost to implement the policy). The goods value field can be equated to ventromedial PFC (vmPFC) and OFC, which according to neurophysiological and clinical studies, have an important role in computation and integration of good values [3, 4, 65]. The action cost is encoded by the “action cost field”. Although it is not clear how action costs are encoded and computed in the brain, recent findings suggest that the anterior cingulate cortex (ACC) is involved in encoding action costs [11, 66, 67]. However, other studies have shown that ACC neurons also encode good values, such as the payoff of a choice and the probability that a choice will yield a particular outcome [68].

The “motor plan formation field” employs a neuronal population code over 181 potential movement directions and is responsible for planning the individual policies towards these directions. Hence, it could be equated with parts of the premotor cortex and parietal cortex, especially the parietal reach region (PRR) and dorsal area 5, for reaches and lateral intraparietal area (LIP) for saccades, which are involved in planning of hand and eye movements, respectively. One of the novelties of the proposed computational framework is that the motor plan formation field dynamically integrates all the decision variables into a single variable named relative desirability, which describes the contribution of each individual policy to the motor decision. Because of this property, the simulated neuronal activity in this field is modulated by decision variables, such as expected reward, probability outcome and action cost. This is consistent with series of neurophysiological studies, which show that the activity of neurons in LIP and premotor dorsal area (PMd) is modulated by the probability that a particular response will result in reward and the relative reward between competing targets, respectively [12, 32].

To reduce the complexity of the framework, we included only some of the main brain areas that are involved in visuomotor tasks and omitted other relevant cortical regions, such as the primary motor cortex (M1), the somatosensory cortex, the supplementary motor areas, as well as subcortical regions such as the basal ganglia. However, important motor and cognitive processes in action-selection, such as the execution of actions and the learning of sensorimotor associations and reward contingencies are implemented using techniques that mimic neural processes of brain areas that are not included in the framework.

### Conclusions

We present a computational framework for dynamically integrating value information from disparate sources in decisions with competing actions. It is based on the concept that decisions between actions are not made in the medial frontal cortex through an abstract representation of values, but instead they are made within the sensorimotor cortex through a continuous competition between potential actions. By combining dynamic neural field theory with stochastic optimal control theory, we provide a principled way to understand how this competition takes place in the cerebral cortex for a variety of visuomotor decision tasks. The framework makes a series of predictions regarding the cell activity in different cortical areas and the choice/motor behavior, suggesting new avenues of research for elucidating the neurobiology of decisions between competing actions.

## Methods

This section analytically describes the computational framework developed in this study to model the behavioral and neural mechanism underlying decisions between multiple potential actions.

### Dynamic neural fields

DNF models simulate the activity of a network of neurons over a continuous space with a fixed connectivity pattern of local excitation and surrounding inhibition. Instead of some anatomically defined space, DNF models are defined over the space that is spanned by the parameters of the tasks. They are based on the concept of neuronal population coding, in which the values of task parameters, such as the location of a stimulus or movement parameters, are determined by the distribution of the neuronal activity within a population of neurons. Activation in DNFs is distributed continuously over the space of the encoded parameter and evolves continuously through time under the influence of external inputs, local excitation and lateral inhibition interactions as described by Equation (3):

$$\begin{array}{c} \tau \dot{u}\left(x,t\right)=-u\left(x,t\right)+h+S\left(x,t\right)+\int w\left(x-x^{'}\right)\;f\;\left[u\left(x^{'},t\right)\right]dx^{'} \end{array}$$ (3)

where *u*(*x*, *t*) is the local activity of the neuronal population at position *x* and time *t*, and $\dot{u}(x,t)$ is the rate of change of the activation field over time scaled by a time constant *τ*. In the absence of any external input *S*(*x*, *t*), the field converges over time to the resting level *h* from the current level of activation. The interactions between the neurons in the field are defined through the kernel function *w*(*x* − *x* ^′^), which consists of local excitatory and global inhibitory components, Equation (4):

$$\begin{array}{c} w\left(x-x^{'}\right)=c_{exc}e^{-\frac{{\left(x-x^{'}\right)}^{2}}{2\sigma_{exc}^{2}}}-c_{inh}e^{-\frac{{\left(x-x^{'}\right)}^{2}}{2\sigma_{inh}^{2}}} \end{array}$$ (4)

where *c*_*exc*_, *c*_*inh*_, *σ*_*exc*_, *σ*_*inh*_ describe the amplitude and the width of the excitatory and the inhibitory parts of the kernel function, respectively. In this study we used a narrow Gaussian kernel for the excitatory interactions and a broader Gaussian kernel for the inhibitory interactions, such that neurons with similar tuning curves co-excite one another, whereas neurons with dissimilar tuning curves inhibit one another. The only fields with competitive interactions in this study were the reach and saccade motor plan formation DNFs; the other fields had *c*_*exc*_ and *c*_*inh*_ set to zero and therefore combined and encoded their inputs without performing further computation.

The kernel function is convolved with a sigmoidal transformation of the field activity *f*[*u*(*x* ^′^, *t*)], such that only neurons with an activity level that exceeds a threshold participate in the intrafield interactions, Equation (5):

$$\begin{array}{c} f\left(u\left(x\right)\right)=\frac{1}{1+e^{-\beta \left(u(x)\right)}} \end{array}$$ (5)

where *β* controls the steepness of the sigmoid function.

Besides the context cue field, the fields consist of 181 neurons and their spatial dimension spans the circular space between 0° and 180°. The context cue field consists of 100 neurons, in which half of them respond to a saccade cue and half of them respond to a reach cue. The following sections describe how we integrate dynamic neural field theory with stochastic optimal control theory to develop a computational framework that can explain both neural and behavioral mechanisms underlying a wide variety of visuomotor decision tasks.

### Action selection for decision tasks with competing alternatives

Stochastic optimal control theory has proven quite successful at modeling goal-directed movements such as reaching [26], grasping [69] and saccades [70]. It involves solving for a policy *π* that maps states onto actions **u**_*t*_ = *π*(**x**_*t*_) by minimizing a loss function penalizing costly actions (i.e., effort) and deviations (i.e., accuracy) from the goal. Despite the growing popularity of stochastic optimal control models, the preponderance of them are limited only to single goals. However, real environments present people and animals at any moment with multiple competing options and demands for actions. It is still unclear how to define control policies in the presence of competing options.

In the current study we decompose the complex problem of action selection with competing alternatives into a mixture of optimal control systems that generate policies *π* ^′^ *s* to move the effector towards specific directions. The core component of the present framework is the “motor plan formation” DNF that integrates value information from disparate sources and plans the movements to acquire the targets. Each neuron in this DNF is linked with a stochastic optimal control system. When the activity of this neuron exceeds a threshold *γ* at the current state **x**_*t*_, the controller suggests an optimal policy π* that results in a sequence of actions (**u**_*t*_ = π*(**x**_*t*_) = [*u*_*t*_, *u*_*t*+1_, *u*_*t*+*T*_]) to drive the effector from the current state towards the preferred direction of the neuron from a period of time *T*. Note that the policy *π* is related to the preferred direction of the neuron and not to the location of the target. This is the main difference between the optimal control module used in the present framework and classic optimal control studies. However, the mathematical formulation of the optimal control theory requires defining an “end” (i.e., goal) state. In the present framework any “active” controller *i* generates a sequence of actions to move in the preferred direction of the neuron *ϕ*_*i*_ for distance *r*—where *r* is the distance between the current location of the effector and the location of the stimulus in the field encoded by that neuron. For instance, let’s consider a scenario in which a target is located at a distance *r* from the current hand location. The control schema with a preferred direction *ϕ*_*i*_ will suggest an optimal policy $\pi_{i}^{*}$, which is given by the minimization of the loss function in Equation (6):

$$\begin{array}{c} J_{i}\left(x_{t},\pi_{i}\right)={\left(x_{T_{i}}-Sp_{i}\right)}^{T}Q_{T_{i}}\left(x_{T_{i}}-Sp_{i}\right)+\sum_{t=1}^{T_{i}-1}\pi_{i}{\left(x_{t}\right)}^{T}R\pi_{i}\left(x_{t}\right) \end{array}$$ (6)

where *π*_*i*_(**x**_*t*_) is the policy for time instances *t* = [*t*_1_, *t*_2_, …, *T*_*i*_] to move the effector towards the *ϕ*_*i*_ direction; *T*_*i*_ is the time-to-arrive at the position **p**_*i*_; **p**_*i*_ is goal-position of the effector, i.e., the position that the effector is planned to arrive at the end of the movement and is given as: **p**_*i*_ = [*rcos*(*ϕ*_*i*_), *rsin*(*ϕ*_*i*_)]; **x**_*T*_*i*__ is the state vector at the end of the movement; *S* matrix picks out the actual position of the effector and goal-position **p**_*i*_ at the end of the movement from the state vector. Finally, *Q*_*T*_*i*__ and *R* define the precision- and the control- dependent cost, respectively (see S1 text for more details).

The first term of the loss function in Equation (6) determines the current goal of the controller, which is related to the neuron with preferred direction *ϕ*_*i*_ i.e., to move the effector at a distance *r* from the current location, towards the preferred direction *ϕ*_*i*_. The second term is the motor command cost (i.e., the action cost) that penalizes the effort required to move the effector towards this direction, for *T*_*i*_ time-steps.

For a DNF of 181 neurons, each of them with a preferred direction between 0 and 180 deg., we construct a simple loss function using an indicator variable *ν*(**x**_*t*_). This variable encodes the overall relative desirability of each policy with respect to the alternative options—in other words, it categorizes the state space into regions, where following one of the policies is the best option. We can write the loss function as a *ν*-weighted mixture of individual loss functions *J*_*i*_’s, Equation (7):

$$\begin{array}{c} J=\sum_{i=0}^{N}\nu_{i}\left(x_{t}\right)J_{i}\left(x_{t},\pi_{i}\right) \end{array}$$ (7)

where *N* is the number of neurons in the DNF (i.e., 181) and *ν*_*j*_(**x**_*t*_) is the indicator variable associated with the controller *j*. When there is no uncertainty as to which policy to follow at a given time—i.e., only the activity of a single neuron exceeds the threshold *γ*—the *ν*-weighted loss function in Equation (7) is equivalent to Equation (6) with *ν*_*i*_(**x**_*t*_) = 1 for the best current direction, and *ν*_*j* ≠ *i*_(**x**_*t*_) = 0 for the rest of the alternative options. However, when more than one neuron is active, there is uncertainty about which policy to follow at each time and state. In this case, the framework follows a weighted average of the individual policies *π*_*mix*_ to move the effector from the current state to a new one, Equation (8):

$$\begin{array}{c} \pi_{mix}\left(x_{t}\right)=\sum_{i=1}^{M}\nu_{i}\left(x_{t}\right)\;\;\underset{\pi_{i}}{\mathrm{argmin}}J_{i}\left(x_{t},\pi_{i}\right)=\sum_{i=1}^{M}\nu_{i}\left(x_{t}\right)\pi_{i}^{*}\left(x_{t}\right) \end{array}$$ (8)

where *M* is the number of neurons that are currently active, $\pi_{i}^{*}\left({\text{x}}_{t}\right)$ is the optimal policy to move in the preferred direction of the *i*th-neuron from the current state **x**_*t*_, and *ν*_*i*_ is the relative desirability of the optimal policy $\pi_{i}^{*}$ that determines the contribution of this policy to the weighted mixture of policies. For notational simplicity, we omit the * sign, and from now on *π*_*i*_(**x**_*t*_) will indicate the optimal policy related to neuron *i*.

To handle contingencies such as moving targets, perturbations, and the effects of noise, the framework employs a widely used strategy in optimal control theory known as “receding horizon” [71]. According to this strategy, the framework implements only the initial portion of the sequence of actions generated by *π*_*mix*_(**x**_*t*_) for a short period of time *k* (*k* = 10 in this study) and then recomputes the individual optimal policies *π*_*i*_(**x**_*t*+*k*_) from time *t* + *k* to *t* + *k* + *T*_*i*_ and remixes them. This strategy continues until the effector arrives at the selected target.

### Computing policy desirability

We focus next on computing the weights *ν* ^′^ *s* by combining information from disparate sources. Recall that each policy *π*_*i*_ is associated with a cost *V*_*π*_*i*__(**x**_*t*_), which represents the action cost—i.e., cost that is expected to accumulate while moving from the current state **x**_*t*_ in the direction *ϕ*_*i*_ under the policy *π*_*i*_. The cost to implement each individual policy is represented by the “action cost” field, such that the higher the activity of the neurons in this field, the higher the action cost to move in the preferred direction of the neurons. Therefore, the output activity of this field **u**_*cost*_ is projected to the “motor plan formation” field through one-to-one inhibitory connections in order to suppress the activity of neurons with “costly” preferred directions at a given time and state.

However, in a natural environment the alternative options are usually attached with different values that should be incorporated in the decision process. The present framework uses the “goods value” field to encode the good values (e.g., reward, outcome probability) associated with the available options. In the current study we assume that the goods value field represents the expected reward of the available goals, although it is straightforward to extend the field to encode other goods-related features. The expected reward is represented in a two-dimensional neural field in which each neuron is selective for a particular goal position in an allocentric reference frame, **U**_*reward*_. For each effector (eye and hand in these simulations), its position is used to convert activity in this two-dimensional field into a one-dimensional neural field encoding an effector-centered representation of the goods value of each goal. The output activity of each effector-centered field, **u**_*reward*_, projects to the corresponding motor plan formation field with one-to-one excitatory connections. It thus excites neurons in each motor plan formation field that drive the effector towards locations with high expected rewards. One of the novelties of the present framework is that the weights of the connections between the goods value field and the motor plan formation field are plastic and are modified using a simple reinforcement learning rule.

Several of the tasks simulated in the current study require the use of a particular effector depending on a visual cue. The model includes a “context cue field” with neurons which respond noisily to the presence of one of the cues in the task. We used a context cue field with 100 neurons, half of which responded to the reach cue and the rest responded to the saccade cue. The output of this field, **u**_*cue*_, projects to each of the motor plan formation fields with one-to-all excitatory connections. Once the weights of these connections have been learned (see “Sensorimotor association learning in effector choice tasks” section), the field excites all neurons in the motor plan formation field corresponding to the cued effector.

Finally, each motor plan formation DNF receives visual input encoding the direction of each goal in effector-centered coordinates. This is provided by the “stimulus input” DNFs whose neurons had Gaussian tuning curves and preferred directions from 0 to 180 degrees. The output of this field, **u**_*vis*_, projected to the corresponding motor plan formation DNF via one-to-one excitatory connections.

The input to the motor plan formation DNF for each effector *S*_*motor*_(*f*), is a sum of the outputs of the fields encoding the visual stimulus, cues, estimated cost, and expected reward, corrupted by additive noise *ξ* which follows a Gaussian distribution:

$$\begin{array}{c} S_{motor}\left(f\right)=\eta_{vis}u_{vis}\left(f\right)+\eta_{cue}W_{cue}\left(f\right)u_{cue}-\eta_{cost}u_{cost}\left(f\right)+\eta_{reward}u_{reward}\left(f\right)+\xi \end{array}$$ (9)

The parameters *η*_*vis*_, *η*_*cue*_, *η*_*cost*_, and *η*_*reward*_ scale the influence of the input stimulus, cue, cost, and expected reward inputs, respectively. While some studies attempt to find values for these parameters that capture the tradeoff subjects make between cost and reward [72, 73], we set them empirically in order to allow the model to successfully perform the task (see S1 Table, S2 Table, S3 Table and S4 Table in the supporting information for the values of the model parameters used in the current study).

For simulations of tasks using multiple effectors, each effector had its own copy of the cue weights and motor plan formation, cost, and effector-centered goods value fields. Competition between effectors was implemented via massive all-to-all inhibitory connections between their motor plan formation fields:

$$\begin{array}{ccc} S_{motor}\left(f\right) & = & \eta_{vis}u_{vis}\left(f\right)+\eta_{cue}W_{cue}\left(f\right)u_{cue}+\eta_{cost}u_{cost}\left(f\right)+\eta_{reward}u_{reward}\left(f\right)-\\ & & \eta_{effector}\sum_{g\neq f}\sum u_{motor}\left(g\right)+\xi \end{array}$$ (10)

where *η*_*effector*_ scales the inhibitory influence of the motor plan formation DNFs on each other.

## Supporting Information

S1 Fig The model architecture designed to simulate effector choice tasks with single or multiple targets. We extended the present computational theory to model effector choice tasks by duplicating the architecture of the framework and designating one network for saccades and one for reaches. Input to the saccade network is encoded in eye-centered coordinates, whereas input to the reach network is encoded in hand-centered coordinates. We call the motor plan formation DNFs for hand and eye movements the reach field and saccade field, respectively. The reach field receives inhibitory projections from every neuron in the saccade field and vice-versa, implementing the competitive interactions between potential saccade and reach plans, as reported by neurophysiological studies [29, 36]. We also introduced the context cue field that encodes the task context, with half of its neurons responding to the saccade cue (i.e., red cue) and half responding to a reach cue (i.e., green cue). This layer was fully connected with both reach and saccade fields, with weights initially randomized with low random values and trainable through reinforcement learning (see main manuscript for more details).
(TIF)

S2 Fig Effects of reward expectancy on the neural activity of the reach DNF for single target trials. Let’s consider a two-target trials scenario with unequal rewards, such as EV(left target) = 3EV(right target), where EV(.) denotes “expected reward”. Panel **A** illustrates the time course of the average activity of the two neuronal ensembles tuned to the two targets, in a trial in which the higher valued target was selected. Notice that the competition is resolved (i.e., neural activity exceeds the action threshold *γ*) almost immediately after the target onset. Panel **B** depicts an infrequent trial, in which the lower valued target (i.e., right target) wins the competition. Notice that it takes considerably more time to solve the competition, resulting in slower movement time. For more details, see the section “The effects of decision variables on action selection” in the main manuscript.
(TIF)

S1 Text Stochastic optimal control theory. A detailed description of the stochastic optimal control theory used to model reach and eye movements to single targets.
(PDF)

S1 Table Model parameters. The values of the model parameters used in the simulations.
(PDF)

S2 Table Stimulus input field parameters. The values of the stimulus input field parameters used in the simulations.
(PDF)

S3 Table Expected reward field parameters. The values of the expected reward (i.e., goods field) parameters used in the simulations.
(PDF)

S4 Table Motor plan formation field parameters. The values of the motor plan formation field parameters used in the simulations.
(PDF)
