# Supplementary figures and images for: A Biologically Plausible Computational Theory for Value Integration and Action Selection in Decisions with Competing Alternatives
Source: PLoS Comput Biol. 2015 Mar 24;11(3):e1004104. doi: 10.1371/journal.pcbi.1004104 (PMC4372613; doi:10.1371/journal.pcbi.1004104)

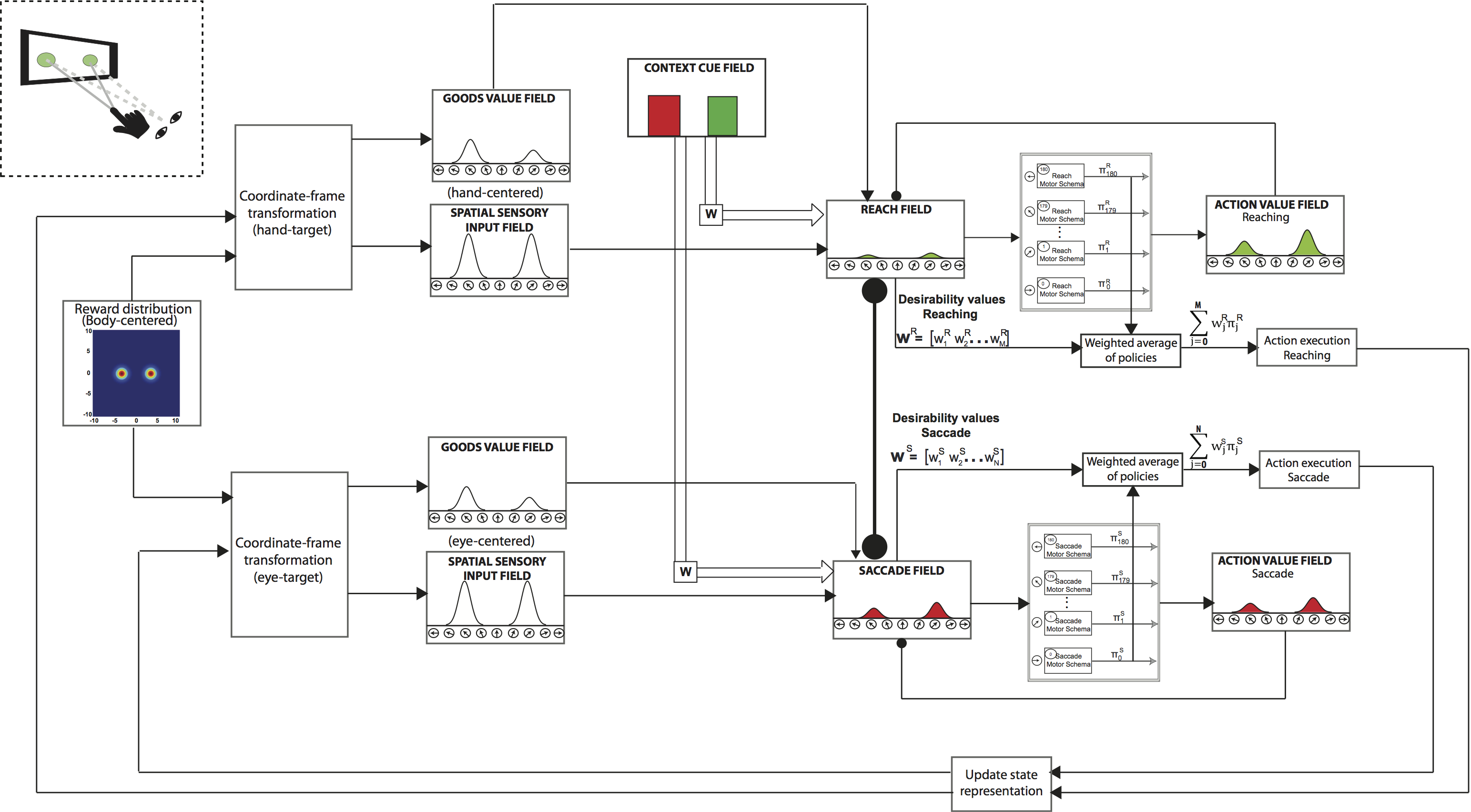

Supplement: S1 Fig — We extended the present computational theory to model effector choice tasks by duplicating the architecture of the framework and designating one network for saccades and one for reaches. Input to the saccade network is encoded in eye-centered coordinates, whereas input to the reach network is encoded in hand-centered coordinates. We call the motor plan formation DNFs for hand and eye movements the reach field and saccade field, respectively. The reach field receives inhibitory projections from every neuron in the saccade field and vice-versa, implementing the competitive interactions between potential saccade and reach plans, as reported by neurophysiological studies [29, 36]. We also introduced the context cue field that encodes the task context, with half of its neurons responding to the saccade cue (i.e., red cue) and half responding to a reach cue (i.e., green cue). This layer was fully connected with both reach and saccade fields, with weights initially randomized with low random values and trainable through reinforcement learning (see main manuscript for more details). (TIF) [file pcbi.1004104.s001.tif]

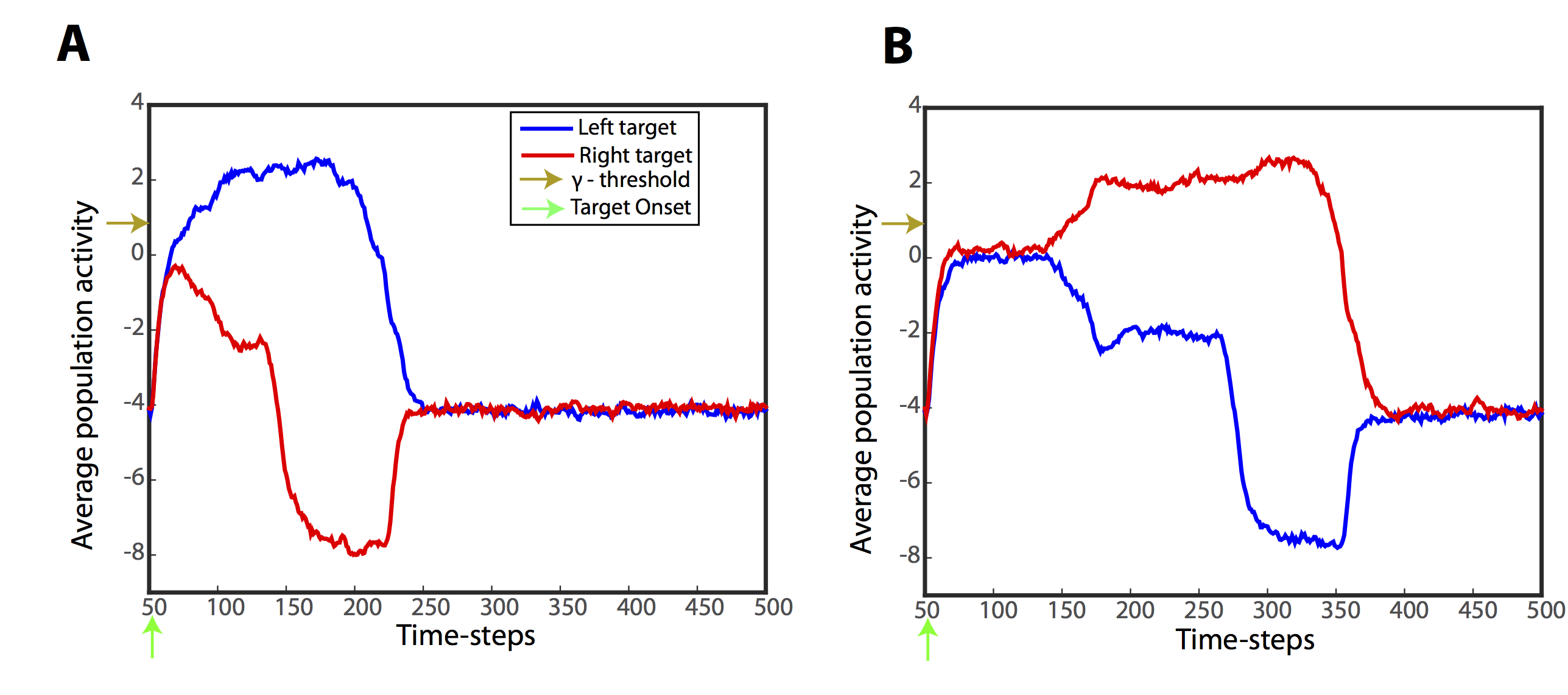

Supplement: S2 Fig — Let’s consider a two-target trials scenario with unequal rewards, such as EV(left target) = 3EV(right target), where EV(.) denotes “expected reward”. Panel A illustrates the time course of the average activity of the two neuronal ensembles tuned to the two targets, in a trial in which the higher valued target was selected. Notice that the competition is resolved (i.e., neural activity exceeds the action threshold γ) almost immediately after the target onset. Panel B depicts an infrequent trial, in which the lower valued target (i.e., right target) wins the competition. Notice that it takes considerably more time to solve the competition, resulting in slower movement time. For more details, see the section “The effects of decision variables on action selection” in the main manuscript. (TIF) [file pcbi.1004104.s002.tif]
