# Supplementary material for: A Biologically Plausible Computational Theory for Value Integration and Action Selection in Decisions with Competing Alternatives
Source: PLoS Comput Biol. 2015 Mar 24;11(3):e1004104. doi: 10.1371/journal.pcbi.1004104 (PMC4372613; doi:10.1371/journal.pcbi.1004104)
Supplement: S1 Text — A detailed description of the stochastic optimal control theory used to model reach and eye movements to single targets. (PDF) [file pcbi.1004104.s003.pdf]

# Text S1

In text S1, we provide details for the stochastic optimal control framework used to model reach and eye movements.

## 1 Hand modeling.

To preserve interpretability, it is important to use a model no more complex than absolutely necessary. In the current study, we modeled the dynamics of the hand as a "point of mass" (m = 1 Kg), in Cartesian coordinates, with 2-dimensional position $\mathbf{p}_t^h = [x_h(t), y_h(t)]^T$ and velocity $\dot{\mathbf{p}}_t^h = [\dot{x}_h(t), \dot{y}_h(t)]^T$ [1]. The combined actions of all muscles is represented by the force vector $\mathbf{f}_t = [f_x(t), f_y(t)]^T$. The motor command $\mathbf{u}_t$ is transformed into these forces $\mathbf{f}_t$ through second-order muscle-like low-pass filters with constants $\tau_1$ and $\tau_2$, and by adding control-dependent multiplicative noise [1].

$$\tau_1\tau_2\ddot{\mathbf{f}}_t + (\tau_1 + \tau_2)\dot{\mathbf{f}}_t + \mathbf{f}_t = \mathbf{u}_t \tag{1}$$

The second-order low pass filters can be written as a pair of coupled first-order filters with outputs $\mathbf{g}$ and $\mathbf{f}$, as:

$$\tau_1\dot{\mathbf{g}}_t + \mathbf{g}_t = \mathbf{u}_t, \quad \tau_2\dot{\mathbf{f}}_t + \mathbf{f}_t = \mathbf{g}_t \tag{2}$$

Given that $\mathbf{p}^G = [x^G, y^G]$ is the position that the hand is planned to arrive, at the end of the movement (i.e., the current goal of the task), the discrete-time state is described by the following $10^{th}$-dimensional vector $\mathbf{x}_t$.

$$\mathbf{x}_t = [x_h(t), y_h(t), \dot{x}_h(t), \dot{y}_h(t), f_x(t), f_y(t), g_x(t), g_y(t), x_G, y_G]^T \tag{3}$$

The discrete-dynamics of the hand are given by Eq. (4):

$$\begin{aligned}
\mathbf{p}^h_{t+\delta t} &= \mathbf{p}^h_t + \dot{\mathbf{p}}^h_t \delta t \\
\dot{\mathbf{p}}^h_{t+\delta t} &= \dot{\mathbf{p}}^h_t + \mathbf{f}_t \frac{\delta t}{m} \\
\mathbf{f}_{t+\delta t} &= \mathbf{f}_t \left(1 - \frac{\delta t}{\tau_2}\right) + \mathbf{g}_t \frac{\delta t}{\tau_2} \\
\mathbf{g}_{t+\delta t} &= \mathbf{g}_t \left(1 - \frac{\delta t}{\tau_1}\right) + \mathbf{u}_t \left(1 + \sigma_c \epsilon_t\right) \frac{\delta t}{\tau_1}
\end{aligned} \tag{4}$$

where $\delta t = 0.01s$ is the sampling period of discretization. In the current framework, we assume that the motor control signal $\mathbf{u}_t$ is corrupted by multiplicative noise described by the product term $\sigma_c \epsilon_t \mathbf{u}_t$, with $\sigma_c = 1$, which is a unitless variable defined as the noise magnitude related to the control signal magnitude [1].

## 2 Reaching to a single goal.

In the current study, each controller is linked with a neuron $i$ from the “motor plan formation field”. The purpose of the controller is to generate an optimal policy that drives the hand to a distance $r$ from the current location, towards the preferred direction $\phi$ of the neuron $i$. Within the stochastic

optimal control framework, control policies originate as the solution of an optimization problem. The basic idea is to find a sequence of motor commands that acquire as much reward as possible, while spending as little effort as possible. For reaching tasks, given the kinematics of the hand and the sensory and motor noise in estimating and controlling the state of the hand, the stochastic optimal control framework finds an optimal policy $\mathbf{u}^*$, for time instances $t = [t_1, t_2, \cdots t_T]$ that optimizes the cost function $J$ described in Eq. (5), for each state of the hand and the environment $\mathbf{x}_t$.

$$J(\mathbf{x}_t, \pi) = \|\mathbf{p}_T^h - \mathbf{p}^G\|^2 + \|\dot{\mathbf{p}}_T^h\|^2 + \|\mathbf{f}_T\|^2 + \sum_{t=1}^{T} \pi(\mathbf{x}_t)^T R \pi(\mathbf{x}_t) \tag{5}$$

The first 3 terms define that current task of the controller - i.e., drive the hand to the position $\mathbf{p}^G = [rcos(\phi)\ rsin(\phi)]$ (first term) and stop there (second and third term). The last term is the motor command cost (i.e., action cost) that penalizes the effort required to arrive at the position $\mathbf{p}^G$ after time $T$ starting from the current state. The matrix $R = \frac{1}{T} \begin{bmatrix} r_x & 0 \\ 0 & r_y \end{bmatrix}$ is the control-dependent cost of the hand motion in the x and y dimension.

We can write Eq. (5) in the general form of optimal control cost function as follows:

$$J = \left(\mathbf{x}_T - S\mathbf{p}^G\right)^T Q_T \left(\mathbf{x}_T - S\mathbf{p}^G\right) + \sum_{t=1}^{T-1} \pi(\mathbf{x}_t)^T R \pi(\mathbf{x}_t) \tag{6}$$

where $S$ matrix picks out the actual position of the hand and the current goal-position $\mathbf{p}^G$ at the end of the movement, from the state-vector. The time-varying matrix $Q_T$ describes the state-dependent cost and is the zero matrix for any time $t < T$ and is equal to the Hessian matrix of the cost function evaluated at the end of the movement $T$.

To minimize the cost function in Eq. (6), a model of the system dynamics and sensory feedback must be incorporated. A plethora of experimental studies provide evidence that the sensory system uses an internal forward model to predict the next state of the system at time $t+1$, $\hat{\mathbf{x}}_{t+1|t}$, based on the sensory feedback $\mathbf{y}_t$, the current state estimate $\hat{\mathbf{x}}_t$ and the control commands $\mathbf{u}_t$ [2]. This prediction is necessary to overcome control instabilities due to noisy sensors and temporal delays. In the current study, we modeled the hand and the state space using linear dynamics and measurement as a discrete linear system, Eq. (7), considering that the motor commands are corrupted by multiplicative noise, normally distributed with zero mean and standard deviation proportional to the magnitude of the control commands and the state variables [1].

$$\begin{aligned} \mathbf{x}_{t+1} &= A\mathbf{x}_t + B\mathbf{u}_t + \xi_t + C(\mathbf{u}_t)\epsilon_t \\ \mathbf{y}_t &= H\mathbf{x}_t + \omega_t \end{aligned} \tag{7}$$

where $A$, $B$ and $H$ are the actual system dynamics and observation matrices, respectively.

$$A = \begin{bmatrix} 1 & 0 & \delta t & 0 & 0 & 0 & 0 & 0 & 0 & 0 \\ 0 & 1 & 0 & \delta t & 0 & 0 & 0 & 0 & 0 & 0 \\ 0 & 0 & 1 & 0 & \frac{\delta t}{m} & 0 & 0 & 0 & 0 & 0 \\ 0 & 0 & 0 & 1 & 0 & \frac{\delta t}{m} & 0 & 0 & 0 & 0 \\ 0 & 0 & 0 & 0 & 1-\frac{\delta t}{\tau_2} & 0 & \frac{\delta t}{\tau_2} & 0 & 0 & 0 \\ 0 & 0 & 0 & 0 & 0 & 1-\frac{\delta t}{\tau_2} & 0 & \frac{\delta t}{\tau_2} & 0 & 0 \\ 0 & 0 & 0 & 0 & 0 & 0 & 1-\frac{\delta t}{\tau_1} & 0 & 0 & 0 \\ 0 & 0 & 0 & 0 & 0 & 0 & 0 & 1-\frac{\delta t}{\tau_1} & 0 & 0 \\ 0 & 0 & 0 & 0 & 0 & 0 & 0 & 0 & 1 & 0 \\ 0 & 0 & 0 & 0 & 0 & 0 & 0 & 0 & 0 & 1 \end{bmatrix}$$

$$B = \begin{bmatrix} \mathbf{0}_{6\times 2} \\ \begin{matrix} \frac{\delta t}{\tau_1} & 0 \\ 0 & \frac{\delta t}{\tau_1} \end{matrix} \\ \mathbf{0}_{2\times 2} \end{bmatrix}, H = \begin{bmatrix} \mathbf{I}_{6\times 6} \mid \mathbf{0}_{6\times 4} \end{bmatrix} \tag{8}$$

The noise variables $\xi_t$, $\omega_t$ and $\epsilon_t$ are normally distributed variables with zero mean and covariance $\Omega^{\xi} \geq 0$, $\Omega^{\omega} \geq 0$ and $\Omega^{\epsilon} = I$, respectively. $C(\mathbf{u}_t)$ is a scaling matrix for control-dependent

system noise, such as $C(\mathbf{u})\epsilon = \sum_i C_i \mathbf{u}\epsilon^i$, where $\epsilon^i$ is the $i^{th}$ component of the random variable $\epsilon$.

Given the belief about the state at time $t$ and the current task of the controller, the controller will suggest an optimal policy $\pi^*(\mathbf{x}_t) = \mathbf{u}^*$ that minimizes the expected cost function in Eq. (5). This form of optimal control is a modified version of the Linear Quadratic Gaussian (LQG) regulator, since the dynamics of the system are linear, the expected cost function is quadratic and the noise is Gaussian, but with signal-dependent noise [1].

The optimal policy is incorporated into the system model to generate a feedback controller that uses its forward model to make predictions $\hat{\mathbf{y}}_t$ from knowledge of controls, dynamics and sensory measurements. These predictions are combined with actual sensory feedback $\mathbf{y}_t$ using Eq. (9) to update the belief about the state in time $t+1$.

$$\hat{\mathbf{x}}_{t+1|t+1} = (A - BL_t)\hat{\mathbf{x}}_{t+1|t} + K_t\left(\mathbf{y}_t - H\hat{\mathbf{x}}_{t+1|t}\right) \tag{9}$$

where $K_t$ is the Kalman gain at time $t$.

## 3 Eye modeling

In the current study, we modeled the globe and the surrounding tissues of the eye by an elastic element with stiffness $k$ that pulls the eye away from the equilibrium point (x = 0), and a viscous element $\mu$ that resists in this motion (for more details about this eye model see [3]). The one-dimensional dynamics of the eye is given by Eq. (10).

$$m\ddot{x} = -kx - \mu\dot{x} + u \tag{10}$$

where $m$ is the inertia of the eye and $u$ are the motor commands.

We used a simple model to translate motor commands $u$ into forces: $\alpha_1 \dot{f} + \alpha_2 f = u$, where $f$ describes the instantaneous force generated by the extra-ocular muscles to move the eye.

Let's assume that the current goal is to move the eye at the position $\mathbf{p}^G = [x_G, y_G]^T$. The discrete-time state is given by the $8^{th}$-dimensional vector $\mathbf{x}_t$, Eq. (11).

$$\mathbf{x}_t = [x_e(t), \dot{x}_e(t), y_h(t), \dot{y}_e(t), f_x, f_y, x_G, y_G]^T \tag{11}$$

We extended this one-dimensional model of the eye to a more realistic two-dimensional model, in which the discrete-time dynamics of the eye are given by Eq. (12):

$$\begin{aligned} \mathbf{p}^e_{t+\delta t} &= \mathbf{p}^e_t + \dot{\mathbf{p}}^e_t \delta t \\ \dot{\mathbf{p}}^e_{t+\delta t} &= -\delta t \frac{k}{m} \mathbf{p}^e_t + \left(1 - \delta t \frac{\mu}{m}\right) \dot{\mathbf{p}}^e_t + \frac{\delta t}{m} \mathbf{f}_t \\ \mathbf{f}_{t+\delta t} &= \left(1 - \delta t \frac{\alpha_2}{\alpha_1}\right) f_t + \mathbf{u}_t \left(1 + \sigma_c \epsilon_t\right) \frac{\delta t}{\alpha_1} \end{aligned} \tag{12}$$

where $\mathbf{p}^e_t = [x_e(t), y_e(t)]^T$ and $\dot{\mathbf{p}}^e_t = [\dot{x}_e(t), \dot{y}_e(t)]^T$ is the 2-dimensional position and velocity of the eye, respectively. Similarly, with the hand model, the product term $\sigma_c \epsilon_t \mathbf{u}_t$ describes the multiplicative noise added to the control signal $\mathbf{u}_t$, with $\sigma_c = 1$ and $\epsilon_t$ a vector of zero-mean random variables with covariance $\Omega^e = \mathbf{I}$. Additionally, $\delta t = 0.001$s is the sampling period of discretization.

## 4 Saccade to a single goal

Even though saccades are brief in time and sensory information seems to have no role in the control of eye movements, recent findings suggest that saccades are not open-loop movements. Instead, the motor commands that generate saccades benefit from a forward internal model that monitors the motor commands and predicts their consequences [4].

We modeled eye movements within the stochastic optimal control framework using a similar approach with the hand movements presented in a previous section. However, the difference with eye movements is that the sensory feedback does not affect the control of the eye movements. Each controller is linked to a neuron $i$ from the motor plan formation field, and generates a policy that drives the eye towards the preferred direction $\phi$ of the neuron $i$. The cost function is the same as the one used in reaching movements (see Eq. (5)).

The dynamics of the eye can be transformed into the form of Eq. (7), with the following matrices:

$$
A = \begin{bmatrix}
1 & \delta t & 0 & 0 & 0 & 0 & 0 & 0 \\
-\frac{k\delta t}{m} & 1-\frac{\mu\delta t}{m} & 0 & 0 & \frac{1}{m}\delta t & 0 & 0 & 0 \\
0 & 0 & 1 & \delta t & 0 & 0 & 0 & 0 \\
0 & 0 & -\frac{k\delta t}{m} & 1-\frac{\mu\delta t}{m} & 0 & \frac{1}{m\delta t} & 0 & 0 \\
0 & 0 & 0 & 0 & 1-\frac{\alpha_1\delta t}{\alpha_2} & 0 & 0 & 0 \\
0 & 0 & 0 & 0 & 0 & 1-\frac{\alpha_1\delta t}{\alpha_2} & 0 & 0 \\
0 & 0 & 0 & 0 & 0 & 0 & 1 & 0 \\
0 & 0 & 0 & 0 & 0 & 0 & 0 & 1
\end{bmatrix}
$$

$$
B = \begin{bmatrix}
\mathbf{0}_{4\times 2} \\
\begin{matrix} \frac{\delta t}{\alpha_1} & 0 \\ 0 & \frac{\delta t}{\alpha_1} \end{matrix} \\
\mathbf{0}_{2\times 2}
\end{bmatrix} \tag{13}
$$

For a monkey eye, we used time constants $\tau_1 = 0.260$ s and $\tau_2 = 0.012$ s as proposed by Keller [5] and Robinson [6]. These time constants are related with the constants of the system dynamic matrices $A$ and $B$ as follows: $\mu = \tau_1 + \tau_2$, $m = \tau_1\tau_2$. Based on these studies [5,6], we set $k = 1$, $\alpha_1 = 0.004$ and $\alpha_2 = 1$.

## References

1. Todorov, E. *Stochastic optimal control and estimation methods adapted to the noise characteristics of the sensorimotor system*, Neural Comput., 17, 1084-1108 (2005)

2. Diedrichsen, J., Shadmehr, R., & Ivry, R *The coordination of movement. Optimal feedback control and beyond*, Trends Cogn Sci.,14, 31-39 (2010)

3. Haith, A., Reppert, R., & Shadmehr, R. *Evidence for hyperbolic temporal discounting of reward in control of movements.*, J Neurosci. 32, 11727-11736 (2012)

4. Ethier, V., Zee, D., & Shadmehrm R. *Changes in control of saccades during gain adaptation*, J Neurosci. 28, 13929-13937 (2010).

5. Keller, E. *Accommodative vergence in the alert monkey. Motor unit analysis*, Vision Res. 13, 1565-1575 (1973)

6. Robinson, D., Gordon, J., & S. Gordon *A model of the smooth pursuit eye movement system*, Biol Cybern. 55, 43-57 (1986).
