# Supplementary material for: A Biologically Plausible Computational Theory for Value Integration and Action Selection in Decisions with Competing Alternatives
Source: PLoS Comput Biol. 2015 Mar 24;11(3):e1004104. doi: 10.1371/journal.pcbi.1004104 (PMC4372613; doi:10.1371/journal.pcbi.1004104)
Supplement: S1 Table — The values of the model parameters used in the simulations. (PDF) [file pcbi.1004104.s004.pdf]

Model parameters

Parameter	Description	Value
α_{cue}	sensorimotor association learning rate	0.01
α_{reward}	expected reward learning rate	0.005
λ_1	rise time constant of eligibility traces	0.01
λ_2	decay time constant of eligibility traces	0.004
η_{vis}	visual input gain	8.0
η_{cue}	cue input gain	0.1
η_{cost}	cost input gain	-0.1
η_{reward}	expected reward input gain	0.35
$\eta_{effector}$	effector competition input gain	-1.25
γ	action threshold	0.9
γ_{rapid}	action threshold for rapid movements (Figure 3)	0.7
