# Supplementary material for: A Biologically Plausible Computational Theory for Value Integration and Action Selection in Decisions with Competing Alternatives
Source: PLoS Comput Biol. 2015 Mar 24;11(3):e1004104. doi: 10.1371/journal.pcbi.1004104 (PMC4372613; doi:10.1371/journal.pcbi.1004104)
Supplement: S4 Table — The values of the motor plan formation field parameters used in the simulations. (PDF) [file pcbi.1004104.s007.pdf]

Motor plan formation field parameters

Parameter	Description	Value
τ	Time constant	5.0
c_{exc}	Amplitude of excitatory portion of weight kernel	18.0
c_{inh}	Amplitude of inhibitory portion of weight kernel	30.0
σ_{exc}	Width of excitatory portion of weight kernel	5.0
σ_{inh}	Width of inhibitory portion of weight kernel	40.0
h	Resting activity level	-5.0
q	Noise level	0.25
σ_q	Width of noise kernel	5.0
β	Steepness of sigmoid activity function	1.0
